# Multi-modal deep learning and explainable AI for predicting multiple dementia-related neuropathologies from brain MRI, clinical, and genetic data

**DOI:** 10.3389/fneur.2026.1839071

**Published:** 2026-07-27

**Authors:** Tamoghna Chattopadhyay, Rudransh Kush, Rahul H. Ankarath, Pavithra Senthilkumar, Emma J. Gleave, Christopher Patterson, Conor Owens-Walton, Sophia I. Thomopoulos, Sterling C. Johnson, Elizabeth C. Mormino, Duygu Tosun, Timothy J. Hohman, Paul M. Thompson

**Affiliations:** 1 Imaging Genetics Center, Mark and Mary Stevens Neuroimaging and Informatics Institute, Keck School of Medicine, University of Southern California, Marina del Rey, CA, United States; 2 Wisconsin Alzheimer’s Disease Research Center, University of Wisconsin School of Medicine and Public Health, Health Sciences Learning Center, Madison, WI, United States; 3 Department of Neurology and Neurological Sciences, Stanford University School of Medicine, Palo Alto, CA, United States; 4 Wu Tsai Neurosciences Institute, Stanford University School of Medicine, Cogen Facility, Stanford, CA, United States; 5 Department of Radiology and Biomedical Imaging, University of California, San Francisco, San Francisco, CA, United States; 6 Department of Neurology, Vanderbilt University Medical Center, Nashville, TN, United States

**Keywords:** Alzheimer’s disease, deep learning, explainable AI, multimodality, neuropathology prediction, VBM

## Abstract

Alzheimer’s disease and related dementias (ADRD) typically involve multiple, overlapping pathologies—such as amyloid-*β* (Aβ), tau, cerebral amyloid angiopathy (CAA), TDP-43, hippocampal sclerosis, and alpha-synuclein—that complicate diagnosis and treatment. While PET and CSF biomarkers can detect abnormal levels of Aβ and tau, they are invasive, expensive, and not widely available. By contrast, structural magnetic resonance imaging (MRI) offers a non-invasive and scalable alternative, one that is now showing promise for neuropathological prediction when combined with artificial intelligence methods. Prior efforts have largely focused on inferring single pathologies such as abnormal Aβ; however, there is a pressing need for models that can jointly predict multiple co-occurring pathologies. In this work, we develop and evaluate a hybrid deep learning framework that integrates 3D T1-weighted brain MRI with demographic, clinical, and genetic covariates to make inferences, in living individuals, regarding the presence of six ADRD pathologies. The models are trained and tested using autopsy-confirmed neuropathology from individuals who were scanned while they were alive. Based on their strong performance on related tasks, we evaluate two machine learning models: (1) a deep learning algorithm based on a 3D convolutional neural network, a widely used model in computer vision applications, and (2) AutoGluon, an automated machine learning framework that automatically selects an approach for the problem. Each method can use both imaging and non-imaging covariates as inputs. To improve model transparency, we incorporate explainable AI methods—including occlusion sensitivity analysis (OSA), Grad-CAM, and Integrated Gradients (IG)—to interpret the spatial contribution of brain regions to model predictions. Finally, we compare the resulting feature importance maps (‘salience maps’) with traditional voxel-based morphometry (VBM) analyses to assess their biological plausibility. Our findings show the promise of multimodal, interpretable AI approaches for comprehensive, non-invasive profiling of dementia-related pathologies.

## 1 Introduction

Alzheimer’s disease and related dementias (ADRD) represent a rapidly escalating global health crisis, affecting over 55 million individuals worldwide—a number expected to rise to around 153 million by 2050 (1). The societal and economic burden of ADRD is enormous, with global costs expected to increase from $1.3 trillion in 2019 to $14.5 trillion by mid-century, reflecting not only the growing prevalence but also the profound impact on families, healthcare systems, and societies (1). This crisis is compounded by the complex and heterogeneous nature of ADRD, which encompasses a spectrum of neurodegenerative and vascular disorders, including Alzheimer’s disease (AD), Parkinson’s disease (PD), dementia with Lewy bodies (DLB), and cerebrovascular pathologies. The underlying neuropathology is multifactorial, and several proteinopathies often co-occur, along with vascular lesions. Classical hallmark neuropathological changes of Alzheimer’s disease––such as amyloid-*β* (Aβ) plaques (2, 3) and hyperphosphorylated tau tangles (4, 5)––are frequently accompanied by cerebral amyloid angiopathy (CAA) (6), transactive response DNA-binding protein 43 (TDP-43) (7, 8) inclusions, hippocampal sclerosis (HPSC), and Lewy bodies (LB) composed of aggregated alpha-synuclein. Notably, LB, CAA, and TDP-43 pathology are each present in 30–80% of AD cases (9–11), and they contribute to cognitive decline independently of Aβ and tau (12). This pathological overlap complicates clinical diagnosis, confounds the evaluation of single-target therapeutics (13–15), and presents significant challenges for trial stratification and treatment planning.

Current biomarker approaches for ADRD rely heavily on positron emission tomography (PET) imaging and cerebrospinal fluid (CSF) assays, which have advanced research and clinical trial design as they can detect Aβ and tau *in vivo*. Even so, these methods are limited by their high cost, invasiveness, and limited accessibility, making them less practical for routine clinical use and large-scale population screening. Moreover, PET imaging and fluid biomarkers do not capture other key pathologies such as TDP-43, CAA, or synucleinopathies (16), leaving a substantial gap in patient neuropathological assessments. As a result, there is a growing movement to expand dementia classification systems such as the ATN (Amyloid, Tau, Neurodegeneration) framework (17, 18) to include vascular, inflammatory, immune, and synuclein-related biomarkers (19, 20), reflecting the multifactorial nature of ADRD. More inclusive and accessible diagnostic tools are crucial, as current single-target therapies still show only modest efficacy in clinical trials, due to the frequent co-existence of multiple pathologies in the aging brain (12).

Magnetic resonance imaging (MRI) offers a non-invasive, widely available alternative for assessing brain macro and microstructure. MRI can capture neuroanatomical changes associated with disease progression, such as atrophy patterns, white matter hyperintensities, and microbleeds. These imaging features are not specific to individual proteinopathies, but their distribution in the brain can provide valuable information on the cumulative impact of multiple pathologies on brain integrity. The challenge lies in extracting and interpreting subtle, disease-relevant patterns from 3D brain MRI, particularly in the context of co-occurring pathologies. Recent advances in artificial intelligence (AI), especially deep learning, have shown promise in addressing this challenge as they can automatically extract complex features from neuroimaging data (21, 22), discovering patterns associated with specific clinical conditions.

Deep convolutional neural networks (CNNs), which were first developed in the computer vision field, can learn hierarchical representations from MRI and PET images, as a basis for ADRD diagnoses and to predict future cognitive decline. This has led to interest in how well they could be trained to infer specific neuropathological substrates. For example, Kim et al. (23) employed a “2.5-D” CNN to predict Aβ positivity from FDG-PET, achieving 0.75 accuracy and 0.86 area under the curve (AUC). Son et al. (24) and Chattopadhyay et al. (25, 26) have tested 2D and 3D CNN architectures for making diagnostic classifications based on amyloid-PET and structural MRI, respectively, while Wang et al. (27) demonstrated strong pathology-specific classification performance using a 3D ResNet on T1-weighted MRI for AD, vascular dementia, and Lewy Body Dementia (DLB). Tosun et al. (12) developed a multilabel classifier using MRI signatures to identify non-Alzheimer’s co-pathologies, including TDP-43, Lewy body disease (LBD), and cerebral amyloid angiopathy (CAA), achieving accuracies of 84% for TDP-43, 81% for LBD, and up to 93% for CAA in an autopsy-confirmed cohort of patients with late-onset Alzheimer’s disease. Despite these advances, most prior studies have focused on predicting a single pathology or clinical diagnosis, which does not fully reflect the reality of ADRD, where multiple pathologies often co-exist and interact. There remains a critical need for models that can jointly infer a range of neuropathological substrates from multimodal data, including MRI, clinical, and genetic information. Such models could enable non-invasive, *in vivo* prediction of individual pathological profiles, advancing precision diagnostics and paving the way for tailored therapeutic interventions. In research, groups of people with non-AD pathology could also be identified in large-scale biobanks, making it easier to discover risk factors for these pathologies, or even genetic or epidemiological factors that resist them.

The integration of diverse data modalities; imaging, clinical, and genetic; has the potential to improve the accuracy and generalizability of neuropathology prediction models. Hybrid architectures that combine CNNs for image analysis with artificial neural networks (ANNs) for ‘tabular’ data (i.e., numeric data on a person’s age, sex, and clinical scores) have shown promise in related domains, such as cancer prognosis (28), and predicting cardiovascular risk (29) and dementia risk (30). In the context of ADRD, multimodal fusion models can combine complementary information from brain structure, clinical history, and genetic risk factors (e.g., APOE genotype) to improve classification. Automated machine learning (AutoML) frameworks, such as AutoGluon (31), offer robust baselines by automating model selection, ensembling, and hyperparameter tuning. While AutoML has been successfully applied to tabular and 2D imaging data, its application to 3D neuroimaging and multi-label classification (i.e., choosing from one of multiple diagnostic groups) remains limited. Benchmarking custom hybrid models against AutoML baselines is essential for assessing the added value of deep multimodal fusion approaches.

Recent work has increasingly evaluated transformer-based architectures (32) for multimodal dementia modeling, leveraging their ability to integrate heterogeneous data sources and capture long-range dependencies. Such approaches have demonstrated strong performance for clinical diagnosis and disease subtyping when trained on large-scale multimodal datasets. We have previously applied transformer-based models to neuroimaging tasks, including amyloid classification from structural MRI (25, 33). However, despite their flexibility, transformer models often require substantially larger training datasets to generalize reliably, particularly in volumetric imaging contexts (33). In neuropathology-focused studies, where autopsy-confirmed labels are scarce and class imbalance is common, models with stronger inductive biases for spatial structure, such as convolutional neural networks, may offer improved robustness and sample efficiency. Accordingly, the present work focuses on CNN-based and hybrid multimodal architectures that are well-suited to moderate-sized, pathology-validated cohorts while enabling rigorous interpretability analyses.

A major barrier to clinical adoption of AI models in neuroimaging is the lack of interpretability. Clinicians require transparent models that provide insight into the features driving predictions and their alignment with known disease mechanisms. Explainable AI (xAI) techniques, such as occlusion sensitivity analysis (34), gradient-weighted Class Activation Mapping (Grad-CAM) (35), and integrated gradients (36), can generate saliency maps highlighting brain regions (22) that an AI-based classifier is using to make decisions regarding each pathology. Comparing these maps to traditional neuroimaging analyses, such as voxel-based morphometry (VBM) (37), may help to validate the biological plausibility of AI-driven findings and foster trust among clinicians and researchers. The importance of interpretability is further highlighted by recent regulatory and ethical guidelines (38), which emphasize the need for explainable and transparent AI systems in healthcare.

While the field has made strides in single-pathology prediction and the use of deep learning for neuroimaging, several gaps remain. Few studies have leveraged autopsy-confirmed labels for multiple pathologies, limiting the ground truth available for model training and evaluation. Most existing models do not incorporate the full spectrum of clinical and genetic covariates, nor do they address the challenges of class imbalance and missing data inherent in real-world cohorts. Furthermore, the generalizability of AI models across diverse populations and imaging protocols is an ongoing concern, underscoring the need for large, heterogeneous datasets and rigorous external validation. Table 1 summarizes key prior studies that have used brain MRI to predict neuropathologies such as amyloid-*β* (Aβ), tau, and some other related proteinopathies.

**Table 1:** Summary of key studies on predicting neuropathologies such as amyloid-*β* (Aβ), Tau, and some other related proteinopathies from brain MRI data.

Authors/teams	Year	Dataset(s)	Methods used	Performance/outcome	Limitations	Significance
Wu et al. (60)	2023	ADNI amyloid PET/MRI & tau PET/MRI	Hippocampal surface MMS + sparse coding + ridge regression	RMSE: 30.8 (Aβ), 0.37 (tau); improved over conventional biomarkers	Focused on hippocampus features only	Accurate quantitative Aβ and tau burden predictions from MRI
Ghazi et al. (61)	2024	ADNI + Dementia Disease Initiation	Deep learning on T1 MRI + demographics	AUC 0.84 for Aβ positivity prediction	Limited to Aβ prediction	Cost-effective non-invasive Aβ screening
Lew et al. (62)	2023	ADNI multi-center amyloid, tau PET	Deep learning with multimodal MRI + clinical/genetic data	AUC: 0.79 (amyloid), 0.73 (tau), 0.86 (neurodegeneration)	Moderate sample diversity	MRI with deep learning predicts PET biomarkers well
Jack et al. (63)	2013	ADNI longitudinal MRI + PET	MRI volumetrics + PET biomarkers	MRI volume changes correlate with amyloid and tau progression	MRI low sensitivity in early amyloid detection	Foundation for MRI-PET combined biomarker studies
Hanseeuw et al. (64)	2019	Longitudinal PET & cognitive data	Amyloid and tau PET + cognition assessment	Aβ rise followed by tau accumulation linked to cognitive decline	No direct MRI prediction	Sequence of amyloid then tau predicts cognitive decline
Silbert et al. (65)	2003	39 subjects postmortem pathology	Longitudinal MRI volumetrics	Rate of ventricular volume increase predicted AD pathology	Small cohort size	Validated MRI atrophy markers for AD pathology
Ossenkoppele et al. (66)	2021	Tau PET amyloid PET comparison	Comparative imaging biomarkers	Tau PET has higher prognostic value vs. amyloid PET	Tau PET still limited in clinical use	Demonstrates prognostic superiority of tau imaging
Wang et al. (27)	2025	National Alzheimer’s Coordinating Center, ADNI	Multi-label deep learning (DeepSPARE indices, explainable heat maps)	Balanced accuracy: AD = 0.844, VD = 0.839, LBD = 0.623; differentiated co-pathologies *in vivo*	Lower accuracy for LBD; model depends on MRI quality and pathology confirmation	Reveals distinct neuroimaging signatures and improves dementia diagnostic specificity
Tosun et al. (12)	2024	Alzheimer’s Disease Neuroimaging Initiative (ADNI), pathology data	Machine learning, precision medicine using imaging/fluid biomarkers	Identified individuals with non-AD co-pathologies; improved trial stratification	Limited by need of high-quality biomarker and imaging data	Supports better clinical trial design; enables targeted interventions for mixed pathologies
Wang et al. (67)	2025	ADNI + SILCODE	Multimodal machine-learning framework integrating: plasma biomarkers, structural MRI features (regional volumes, cortical thickness/area), diffusion MRI structural connectomes, and genetic risk profiles (APOE genotype & polygenic risk scores); Regression (e.g., random forest) for continuous Aβ prediction.	Baseline model (plasma + clinical): *R*^2^ ~ 0.56 (NeuroImage report); Adding neuroimaging + APOE: *R*^2^ increased to ~0.617–0.63; Replacing APOE with polygenic risk score: *R*^2^ ~ 0.64–0.637.	Non-PET predictions remain surrogate estimates (not replacing amyloid PET fully); generalizability beyond ADNI/SILCODE unknown.	Demonstrates that integrating plasma, imaging, and genetic data improves noninvasive prediction of cerebral amyloid (Aβ) burden compared to plasma alone. Suggests a scalable alternative to costly/invasive PET imaging for early Alzheimer’s risk assessment and cohort stratification.
Long et al. (68)	2022	720 cognitively normal adults (age 42–91) from longitudinal memory/aging cohorts; 57 autopsied cases	Baseline amyloid PET (PIB or AV45) and/or CSF p-tau181/Aβ42 biomarker assessment; longitudinal clinical follow-up (CDR, MMSE); Cox models for progression; comparison with neuropathology at autopsy using standard criteria	Preclinical Alzheimer’s biomarkers strongly predicted progression to cognitive impairment: 34.4% of biomarker-positive vs. 8.4% of biomarker-negative developed impairment; among 57 autopsies, 90.9% of biomarker-positive had AD neuropathology vs. 8.6% of negative; sensitivity ~87%, specificity ~94% for predicting neuropathology	Autopsy sample smaller (57) than overall cohort (limiting some subgroup analyses); results from specialized longitudinal cohort may not generalize to all populations; PET/CSF biomarkers remain surrogate markers (not interventions)	Validates that amyloid PET and CSF biomarkers in preclinical cognitively normal adults can predict both later cognitive impairment and Alzheimer’s neuropathology at autopsy, supporting their use for early detection and risk stratification in research and intervention trials
Xue et al. (56)	2024	Nine independent, geographically diverse cohorts (total ~51,269 participants), including NACC, ADNI, FHS, AIBL, OASIS, PPMI, NIFD, LBDSU, 4RTNI	Multimodal AI/ML framework using transformer-based deep learning integrating demographics, medical history, neuropsychological tests, functional evaluations, and multimodal neuroimaging; classification of 10 dementia etiologies; comparison with traditional clinician assessments	Micro-averaged AUROC ~0.94 for classifying normal cognition, MCI, dementia; ~0.96 for differentiating dementia etiologies; ~0.78 mean AUROC for mixed dementias; AI-assisted clinician AUROC improved ~26% over clinicians alone; alignment with biomarker and postmortem data	Lower macro-average precision for some etiologies; performance varies with incomplete data	Demonstrates that AI using routinely collected multimodal clinical data can differentiate multiple dementia etiologies and augment clinician diagnosis, with implications for screening, management, and trial stratification
Goenka et al. (69)	2021	Review covers multiple datasets from literature (e.g., ADNI, AIBL, OASIS, etc. across reviewed studies)	Survey of deep learning methods applied to Alzheimer’s prediction using biomarkers, especially convolutional neural networks (CNNs), autoencoders, RNNs, GANs and multimodal approaches combining structural MRI, PET, CSF and other biomarkers	Synthesizes findings that multimodal neuroimaging + deep learning generally improves classification accuracy vs. single modality; highlights 3D subject/ROI/patch-level approaches influence performance	Literature review—no new empirical results; heterogeneity of studies limits direct performance comparison; variation in datasets, tasks, and evaluation metrics noted	Comprehensive overview of deep learning advances for Alzheimer’s disease prediction across biomarkers, emphasizing that multimodal models often outperform single-modality methods and identifying open research areas for model validation and clinical acceptance
Gupta et al. (70)	2019	ADNI	Multimodal biomarker integration (FDG-PET, sMRI, CSF proteins, APOE genotype); early feature fusion; dimensionality reduction (Truncated SVD); multiclass SVM classifier with grid search and 10-fold cross-validation	Multimodal model improved classification: AUROC ~98.33% (AD vs. HC), ~96.83% (AD vs. MCIs), ~94.64% (AD vs. MCIc), ~96.43% (HC vs. MCIc), etc.; outperformed unimodal approaches	Relatively small sample with complete multimodal data; no external validation cohort; focused on classification rather than longitudinal prediction; machine learning performance may not generalize outside ADNI structure	Demonstrates that combining genetic, fluid, and imaging biomarkers in an SVM framework significantly improves discriminative performance among AD, MCI subgroups, and healthy controls compared with single biomarker modalities. Supports the value of multimodal integration for early and differential detection of AD/MCI.
Goenka et al. (71)	2023	ADNI: T1w + AV-45 PET Scans	Multimodal deep learning with ensembled volumetric ConvNet models using three neuroanatomical feature extraction approaches: 3D Subject-based, 3D Patch-based, and 3D Slice-based networks	Overall accuracy for 3-class (AD vs. MCI vs. NC): ~93.01% for the 3D-Subject model; Patch-based achieved ~89.55%; Slice-based ~89.37%. Larger patches yielded higher accuracy among patch approaches.	No external validation reported; focus on classification accuracy without longitudinal prediction	Demonstrates high-accuracy multimodal, multi-class Alzheimer’s classification using structural (MRI) and molecular (PET) imaging with 3D deep learning; comparative evaluation of different feature extraction frameworks suggests 3D Subject-level features provide strongest performance among tested designs

In this study, we address these challenges by developing and evaluating deep learning models for the joint prediction of multiple co-occurring neuropathologies; including amyloid-*β* (Aβ), tau, cerebral amyloid angiopathy (CAA), TDP-43, hippocampal sclerosis (HPSC), and Lewy body (LB) pathology; using *in vivo* structural MRI in combination with demographic, clinical, and genetic covariates. Unlike prior work that focuses on single biomarkers or clinical diagnoses, our approach targets pathology-level inference based on autopsy-confirmed labels, enabling direct biological validation of model predictions. Leveraging a large, diverse, multi-center cohort with postmortem neuropathology, we systematically evaluate covariate-only, imaging-only, and multimodal fusion models to establish the added value of MRI for neuropathology prediction. Our custom hybrid architecture integrates volumetric MRI with tabular data and is benchmarked against an automated machine-learning framework (AutoGluon), providing a head-to-head comparison of two distinct multimodal learning paradigms. To enhance biological interpretability, we apply multiple explainable AI techniques and relate CNN-derived salience maps to traditional voxel-based morphometry (VBM) findings. By advancing a multi-target, multimodal, and interpretable AI framework for neuropathology prediction, this work examines the feasibility of non-invasive, individualized characterization of dementia-related disease substrates and lays the groundwork for improved stratification, mechanistic insight, and precision medicine in ADRD.

## 2 Imaging data and preprocessing steps

The primary dataset for our experiments consisted of a subset of the National Alzheimer’s Coordinating Center (NACC) database (39) (N = 950; age: 76.01 ± 11.86, 390F/560M), with rich multimodal data including both *in vivo* MRI imaging and *post mortem* neuropathology. The NACC database, established in collaboration with the National Institute on Aging, brings together standardized longitudinal clinical, genetic, biomarker, imaging, and neuropathology data from over 40 Alzheimer’s Disease Research Centers across the United States and is the largest resource of its kind in the country. Participants in the NACC dataset range from cognitively unimpaired adults to those with mild cognitive impairment (MCI) or clinical dementia. Cognitive status and clinical diagnoses are systematically collected during annual visits using the Uniform Data Set (UDS), which includes extensive neuropsychological assessments, demographic data, genetic information (e.g., APOE status), and, in a subset, MRI and PET imaging, as well as cerebrospinal fluid biomarkers. For deceased participants, a standardized neuropathological evaluation is compiled. The dataset also includes an array of detailed neuropathological assessments performed at autopsy to characterize the presence of variant dementias such as frontotemporal lobar degeneration and Lewy-body dementia.

In our study, we specifically selected cases with high-quality matched MRI and autopsy data. As HPSC and TDP-43 labels were unevenly distributed across diagnostic groups (625 dementia, 204 cognitively normal, 121 MCI), we manually balanced training, validation and test sets in the ratio 80:10:10. We ensured that there was no overlap between training, validation, and test data subsets at the subject level. Subjects with multiple MRI scans were assigned exclusively to a single split (training, validation, or test), and no subject appeared in more than one subset. Additionally, the test dataset contained only one scan per subject. For MRI preprocessing, 3D volumetric T1-weighted (T1w) images were subjected to several rigorous steps (40): nonparametric intensity normalization using N4 bias field correction, skull stripping for precise brain extraction, nonlinear registration to an in-house template with six degrees of freedom, and isometric voxel resampling to 2 mm. This resulted in uniform image dimensions of 91x109x91. The voxel values were then normalized to a 0–1 range using min-max scaling, facilitating downstream analysis.

### 2.1 Choice of neuroimaging modalities

We chose to use T1-weighted MRI for the current study, as it is by far the most widely available and is routinely collected in clinical and research settings. It could be that T2-weighted sequences such as FLAIR, SWI, QSM or diffusion imaging would add greater benefit, however because these were not all collected for participants in the neuropathological dataset, and because they are less commonly collected, we opted to use T1-weighted MRI. A similar logic applies to the use of PET, as a classifier trained using that data modality would be less readily used in clinical practice.

## 3 Models and experiments

### 3.1 Random forest and Shapley values

Random forest models (41) and Shapley value (SHAP) methods (42, 43) were employed as the reference approaches for predicting individual neuropathological outcomes using only covariate data (i.e., not using brain imaging). Random forest analysis is an ensemble machine learning technique that builds multiple decision trees using bootstrapped samples and random subsets of features, providing robust classification and reducing the risk of overfitting, particularly in complex biomedical datasets. This method is well-suited for discovering non-linear relationships and interactions among predictors such as APOE4 allele count, APOE3 allele count, age, sex, diagnosis, and age_delta (defined as the interval between the person’s age at death and the age at their MRI scan), as well as a reduced covariate set omitting genetic markers. In these experiments, the random forest algorithm was separately trained on each neuropathological target, allowing for the assessment of distinct covariate effects for every outcome. The code was implemented using python and the scikit-learn package and will be made available in Github upon acceptance.

Interpretability of feature contributions was achieved by applying Shapley additive explanations (SHAP), a model-agnostic approach rooted in cooperative game theory. SHAP assigns each covariate an importance score, quantifying its contribution to the predicted outcome for an individual subject. By summarizing SHAP values across subjects and experiments, the analysis clarified how variables including APOE genotype, age, and clinical status influenced neuropathological classification. This framework helped to identify meaningful relationships and allowed comparison of feature impact between models including genetic data and those limited to demographic and clinical variables. Model performance was assessed using balanced accuracy and F1 score metrics.

### 3.2 AutoGluon

**Figure 1:**
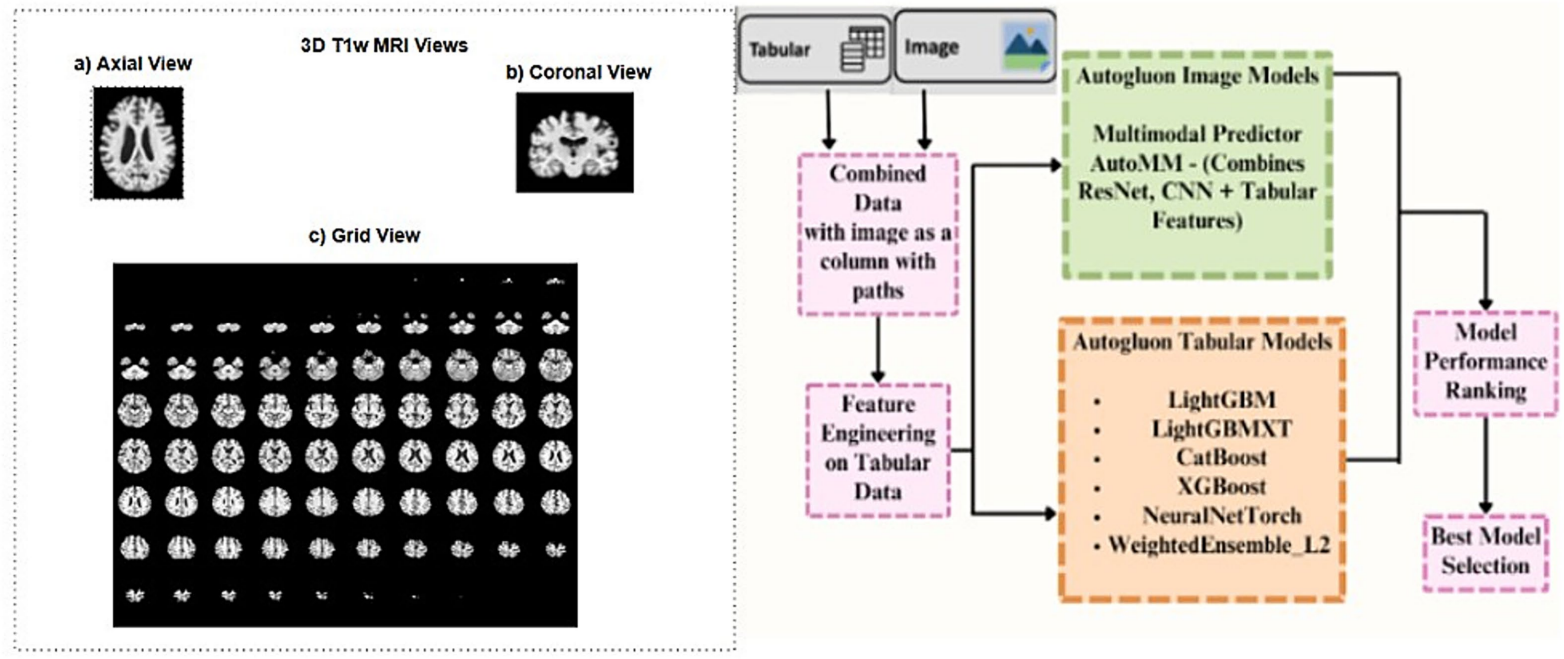
Left: Image inputs for the AutoGluon models: **(a)** axial view, **(b)** coronal view, and **(c)** grid view of 3D T1w MRI slices. Right: AutoGluon architecture, which automatically selects a neural network architecture from a range of traditional models.

We employed AutoGluon, an automated machine learning (AutoML) framework capable of handling multimodal inputs, specifically tabular and image data, with minimal manual tuning. AutoGluon includes streamlined data preprocessing, automatic model selection, and hyperparameter optimization, and its ensemble strategies are designed to be effective for improving generalization and mitigating overfitting. In our experiments, we evaluated three configurations with AutoGluon (Figure 1): first, using only the covariates, mirroring the reference Random Forest model; second, using the entire 3D T1-weighted MRI volumes, reorganized into a grid of 2D slices to preserve comprehensive anatomical detail along with the covariates; and third, extracting the al slices from each T1-weighted scan to reduce computational load while preserving key anatomical features and using it along with the covariates. This diversity of input experiments enabled a thorough comparison of the relative contributions of demographic, clinical, and imaging features to neuropathological prediction. Model performance was assessed using balanced accuracy and F1 score metrics.

### 3.3 3D CNN

**Figure 2:**
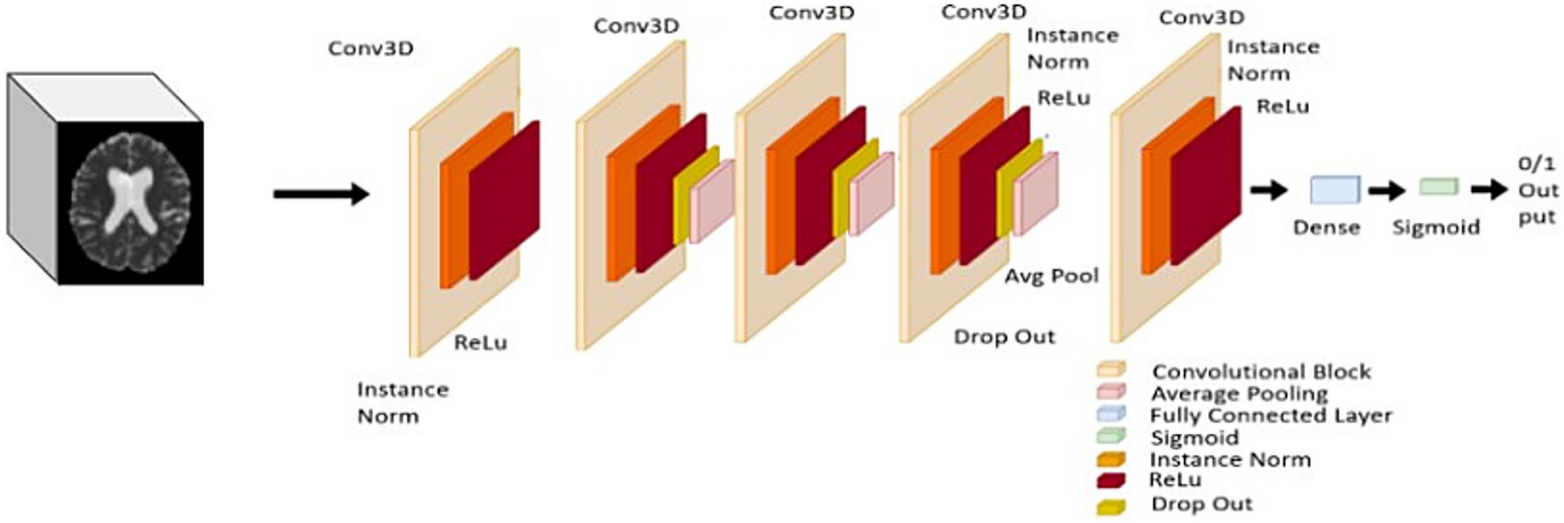
3D CNN Architecture. The model consists of sequential layers that process volumetric brain imaging data. Each “plate” in the diagram represents layers of artificial neurons that collectively form a mathematical function trained to recognize patterns in the data. Convolutional layers extract spatial features by applying filters that scan across the input images. Pooling layers downsample the feature maps, reducing their size while retaining key information. Dropout layers randomly deactivate a subset of neurons during training to prevent overfitting. Dense (fully connected) layers combine learned features to produce the final classification or prediction. This structure enables the network to model increasingly abstract representations of brain anatomy and activity.

The 3D convolutional neural network (CNN) architecture employed for image-only neuropathology classification (Figure 2) consisted of four consecutive 3D convolutional layers with 3 × 3 kernels, capturing local spatial patterns throughout the 3D T1-weighted scans. After the convolutional blocks, a 1 × 1 convolution layer was applied to condense the learned feature maps, followed by a fully connected output for classification. To ensure stable training and support the network’s ability to generalize, instance normalization and ReLU activation functions were used after each layer, promoting effective representation while mitigating internal covariate shift. To further prevent overfitting, dropout regularization with a rate of 0.5 was applied after the second, third, and fourth convolutional layers. Early stopping was implemented during training to halt model updates when validation performance plateaued, preserving model robustness. Dimensionality reduction was achieved by applying 3D average pooling layers with 2 × 2 kernels, reducing computational load and retaining salient features for downstream classification. The network was trained using the Adam optimizer with a learning rate of 1 × 10^−4^, incorporating exponential decay (0.96) to adaptively decrease the learning rate as training progressed. Empirical hyperparameter tuning helped optimize training duration and model parameters; the final setup used a batch size of 8 over 100 epochs. Model performance was assessed using balanced accuracy and F1 score metrics.

For the deep-learning analyses, scanner site and field strength were not explicitly included as model inputs or nuisance variables. Instead, scanner-related variability was addressed implicitly through standardized preprocessing (including intensity normalization, spatial normalization, and resampling to a common voxel resolution) and by training models on a large, heterogeneous, multi-center dataset. This approach encourages learning of features that are robust to acquisition differences but does not involve explicit harmonization. Even so, it cannot be assumed that image harmonization always helps with classification, as it may not preserve the features learned by the classifiers. Because of this, it can be more effective for the classifier to be exposed to a range of scanning protocols in the training phase, rather than to correct the test data.

We also used various xAI models to generate saliency maps highlighting brain regions most relevant to each pathology, as described in the following subsections. Each xAI model has distinct limitations, which is why a comprehensive set of models was included for comparison. For instance, some XAI methods struggle with reliability and consistency, often producing unstable or irreproducible explanations depending on the input or minor perturbations, as revealed by systematic large-scale studies on neuroimaging data (44). Specific models may also yield explanations that are not faithful to the true decision process of the underlying neural network, leading to misleading or incomplete interpretations. Additionally, a number of popular techniques, such as saliency maps, can highlight image features that are actually not relevant or critical to the final model output, thus failing to provide truly informative explanations for practitioners. Given these critical weaknesses, using a single xAI method may not reflect the outputs of current interpretability approaches; therefore, comprehensive evaluation necessitated deploying all available xAI models with our 3D CNN to achieve a fair, generalizable comparison.

#### 3.3.1 Occlusion sensitivity analysis

**Figure 3:**
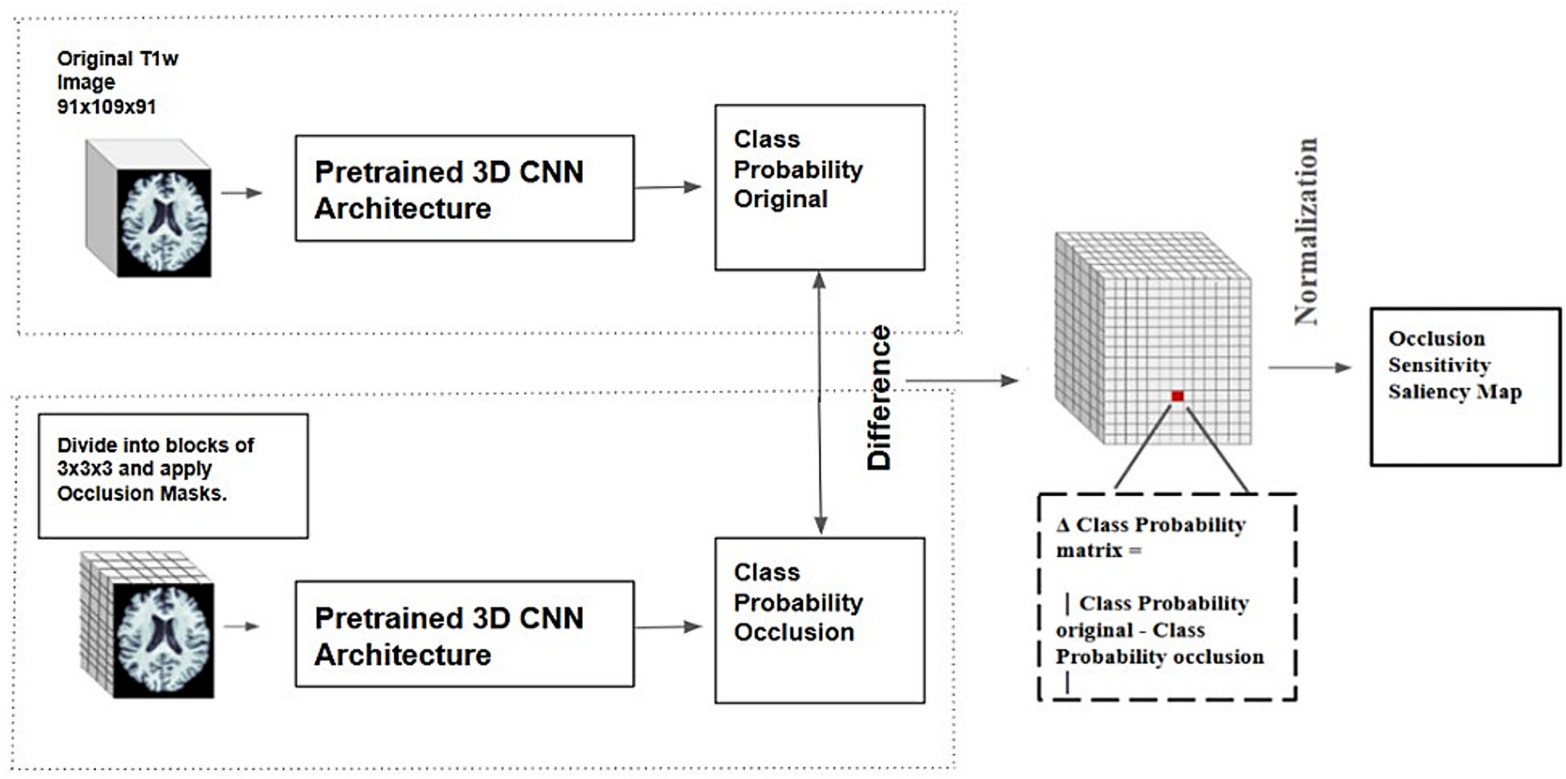
OSA Architecture.

Occlusion sensitivity analysis (OSA) (45) is a perturbation-based technique where parts of the input are intentionally masked to assess how these alterations influence the model’s output. In this study, we employed a cube-shaped mask measuring 3 × 3 × 3 voxels (Figure 3), setting the masked regions to zero. By systematically sliding this mask across the entire brain volume without overlap, we can evaluate how the model’s predictions change. The resulting variation in balanced accuracy, comparing performance with and without occlusion, serves as an indicator of the relative importance of each region. Specifically, we focus on how the classification probability of the selected class (after the final SoftMax layer) is affected. Mapping these variations produces a *saliency map* that highlights the brain regions most influential in the model’s decision-making process. After conducting OSA across 100 test subjects, we generated a voxel-wise averaged map to identify consistent patterns that the model relies on across individuals.

#### 3.3.2 Guided GradCAM

In the Grad-CAM method, the class-specific localization map, $L_{\text{Grad}-\mathrm{CAM}}^{c}$ is generated by integrating the feature maps $A_{k}$ from a chosen convolutional layer with corresponding weights. These weights, denoted as $\alpha_{k}^{c}$ , are determined through global average pooling of the gradients of the target class *c* with respect to each feature map $A_{k}$ . The resulting weighted sum of feature maps is then passed through a ReLU activation function to retain only the positive contributions:


$$L_{\text{Grad}-\mathrm{CAM}}^{c}=\text{ReLU}\;(\sum_{k}\alpha_{k}^{c}\;A_{k})$$

*Guided Grad-CAM* extends this approach by combining the Grad-CAM heatmap with Guided Backpropagation through element-wise multiplication, yielding high-resolution visualizations that are specific to each class. In Guided Backpropagation, only positive gradients are propagated backward, while negative gradients are set to zero during the backward pass through ReLU activations. By merging the spatial localization capability of Grad-CAM with the detailed gradients from Guided Backpropagation, Guided Grad-CAM provides an enhanced and interpretable visualization of deep neural network decisions. We applied the same procedure using Guided Grad-CAM and reported the results as an average group analysis.

#### 3.3.3 Shapley values

SHAP (Shapley additive explanations) provides a pixel-level or voxel-level understanding of how a convolutional neural network (CNN) makes image classification decisions by attributing importance values to each feature in the input, such as individual pixels. In practice, SHAP uses sampling and approximation based on the Shapley value concept from cooperative game theory, estimating how much each pixel contributes to the model’s output for a specific class. The resulting SHAP figures visualize these attributions using a color-coded scheme: red pixels signify regions that increase the likelihood of a particular class being predicted, while blue pixels correspond to regions that decrease this probability. We applied the same procedure using Shapley values and reported the results as an average group analysis.

#### 3.3.4 Smooth grad

Smooth Grad (46) is an interpretability technique designed to produce clearer and more reliable sensitivity maps for neural networks, especially in image classification tasks. Rather than relying solely on raw gradients, which often generate noisy and fragmented saliency maps, Smooth Grad achieves its enhanced explanations by introducing Gaussian noise to the input image multiple times and averaging the resulting gradient-based attribution maps. This process suppresses high-frequency artifacts, highlighting coherent and semantically meaningful regions that influence model predictions. We applied the same procedure using Smooth Grad and reported the results as an average group analysis.

#### 3.3.5 Integrated gradients (IG)

Integrated Gradients (36) is an xAI technique designed to attribute a model’s prediction to individual input features, such as image pixels, by aggregating gradient information along a path from a baseline input to the actual input. Instead of relying on a single gradient evaluation, this method takes multiple incremental steps from the baseline (commonly a black or neutral image) toward the image being explained, calculating gradients at each step to capture a more holistic view of feature importance. The resulting attribution map reveals which regions in the input image contribute most strongly to the model’s decision, producing saliency masks that spotlight the most influential pixels for a given class prediction. When this attribution mask is overlaid on the original image, it highlights the areas the model relied on. We applied the same procedure using Integrated Gradients and reported the results as an average group analysis.

#### 3.3.6 Layer-wise relevance propagation (LRP)

Layer-wise Relevance Propagation (LRP) (47) is an xAI technique designed to interpret the decisions of deep neural networks by decomposing the output prediction back to the input features through a layer-by-layer backward pass. Starting with the model’s prediction, relevance scores are assigned to the output layer neurons and then propagated backward through the network architecture following specific propagation rules, ensuring conservation of relevance at each layer so that the total relevance remains constant from output to input. This process results in heatmaps that indicate how much each input feature, such as pixels in an image, contributed to the final decision. LRP differs from gradient-based methods by explicitly decomposing the prediction rather than relying solely on gradient magnitudes, which often leads to more focused and interpretable explanations. The relevance conservation principle ensures that the sum of relevances at each layer equals the model’s output, providing a theoretically grounded explanation of which parts of the input influenced the predictions the most. We applied the same procedure using LRP and reported the results as an average group analysis.

### 3.4 Voxel-based morphometry (VBM)

**Figure 4:**
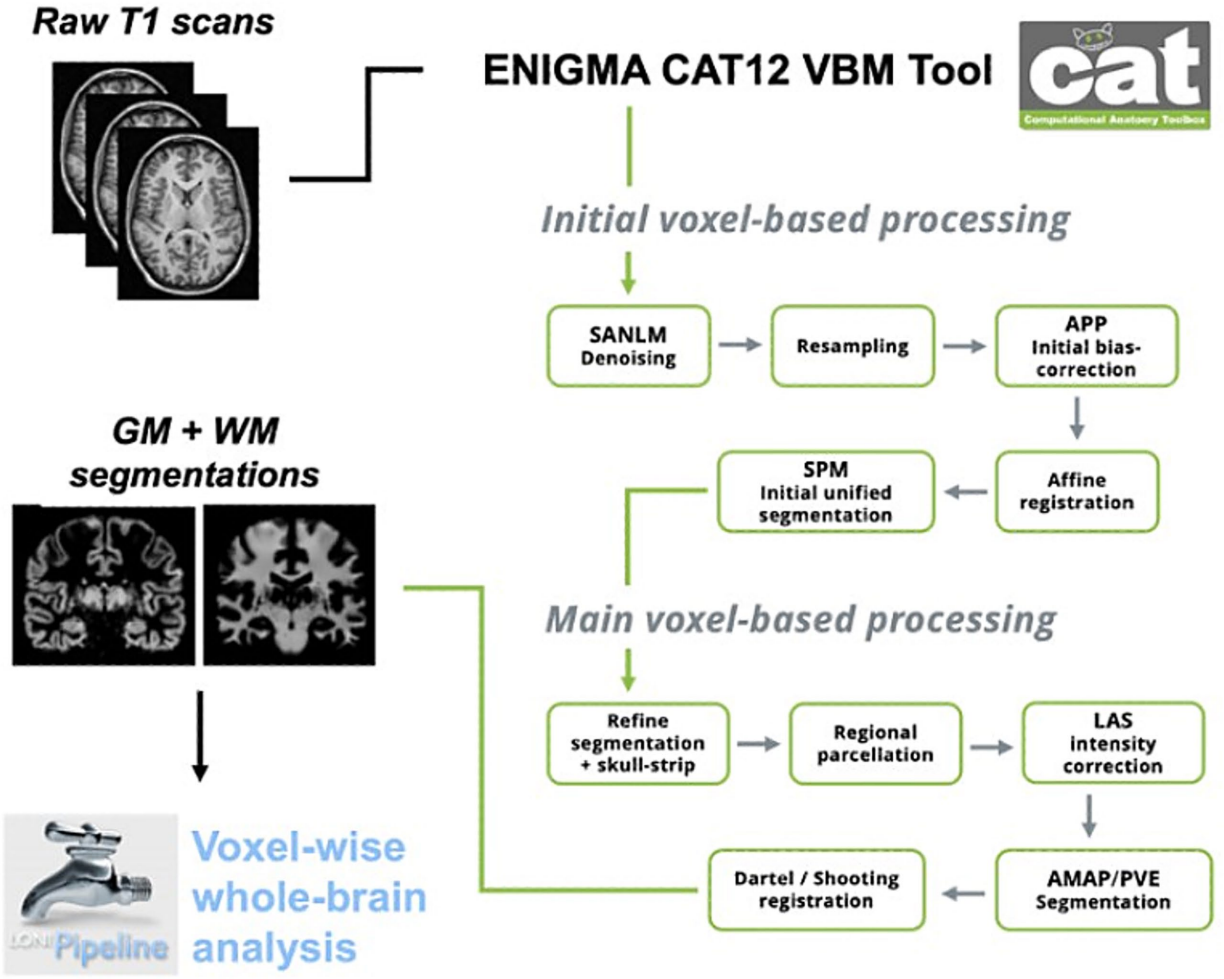
VBM Pipeline and Analysis Workflow. Schematic overview of the ENIGMA CAT12 voxel-based morphometry (VBM) pipeline applied to T1-weighted structural MRI data, illustrating preprocessing, segmentation, registration, and voxel-wise statistical analysis steps. The ENIGMA CAT12 VBM pipeline processes raw 3D T1-weighted MRI scans with a first module including a spatial adaptive non-local means (SANLM) denoising filter, bias correction, segmentation, and registration, followed by a second module for spatial registration including skull-stripping, regional parcellation, intensity transformations and refinement of brain tissue classification and volume estimations for voxel-wise analysis. LAS (Local Adaptive Segmentation) improves local tissue intensity normalization, AMAP (Adaptive Maximum *A Posteriori*) segmentation provides robust tissue classification without relying on population priors, and PVE (Partial Volume Estimation) addresses voxels containing mixtures of different tissues, enhancing segmentation accuracy at boundaries.

Voxel-based Morphometry (37, 48) is an advanced neuroimaging analysis technique that enables the investigation of focal differences in brain anatomy using high-resolution volumetric MRI data. When it was first introduced (37), VBM represented a significant methodological advancement over traditional morphometry approaches, which at the time involved manual delineation of regions of interest. With VBM, automated, whole-brain analysis is possible, without prior selection of specific structures, enabling unbiased assessment of focal or diffuse volumetric abnormalities. In this study, VBM was applied to 512 T1-weighted MRI scans from the NACC cohort (Figure 4), which was a subset of the data used for training the deep learning models. For each subject with multiple scans, only the latest scan was included so that each participant contributed a single data point to the regression analyses, ensuring unbiased statistical testing and independence of observations. This differs from the deep learning dataset, which could include multiple scans per subject to maximize data volume for model training. Thus, the final VBM sample size (N = 512) reflects the maximum number of subjects who met these criteria. Data preprocessing and analysis were performed using the Computational Anatomy Toolbox (CAT12) (48), which provides a robust pipeline for large-scale brain segmentation and morphometric evaluation. The workflow included bias correction, denoising, skull stripping, spatial normalization to a standard template, tissue segmentation into gray matter (GM), white matter (WM), and cerebrospinal fluid (CSF), and smoothing of GM brain masks with a 6-mm Gaussian kernel to optimize signal detection for subsequent voxel-wise analyses.

Following preprocessing, voxel-wise statistical analyses were conducted to determine regional brain volume differences that were statistically associated with relevant clinical and demographic variables. Statistical modeling was implemented using linear mixed effects models within the LONI pipeline (v.7.0.3; utilizing R version 2.9.2) (49), incorporating covariates such as age at imaging, age interval difference (age at death minus age at imaging), sex, educational level, APOE4 allele count, clinical diagnosis (normal cognition, mild cognitive impairment, dementia), total intracranial volume, scanner field strength, and NACC scan site as a random effect. This approach allowed for sensitive mapping of morphometric changes across the entire brain and helped to relate regional GM volume differences to each of the neuropathologies. These maps were then compared to the saliency maps obtained from the various xAI models used for the 3D CNN architecture.

### 3.5 Hybrid CNN architecture

**Figure 5:**
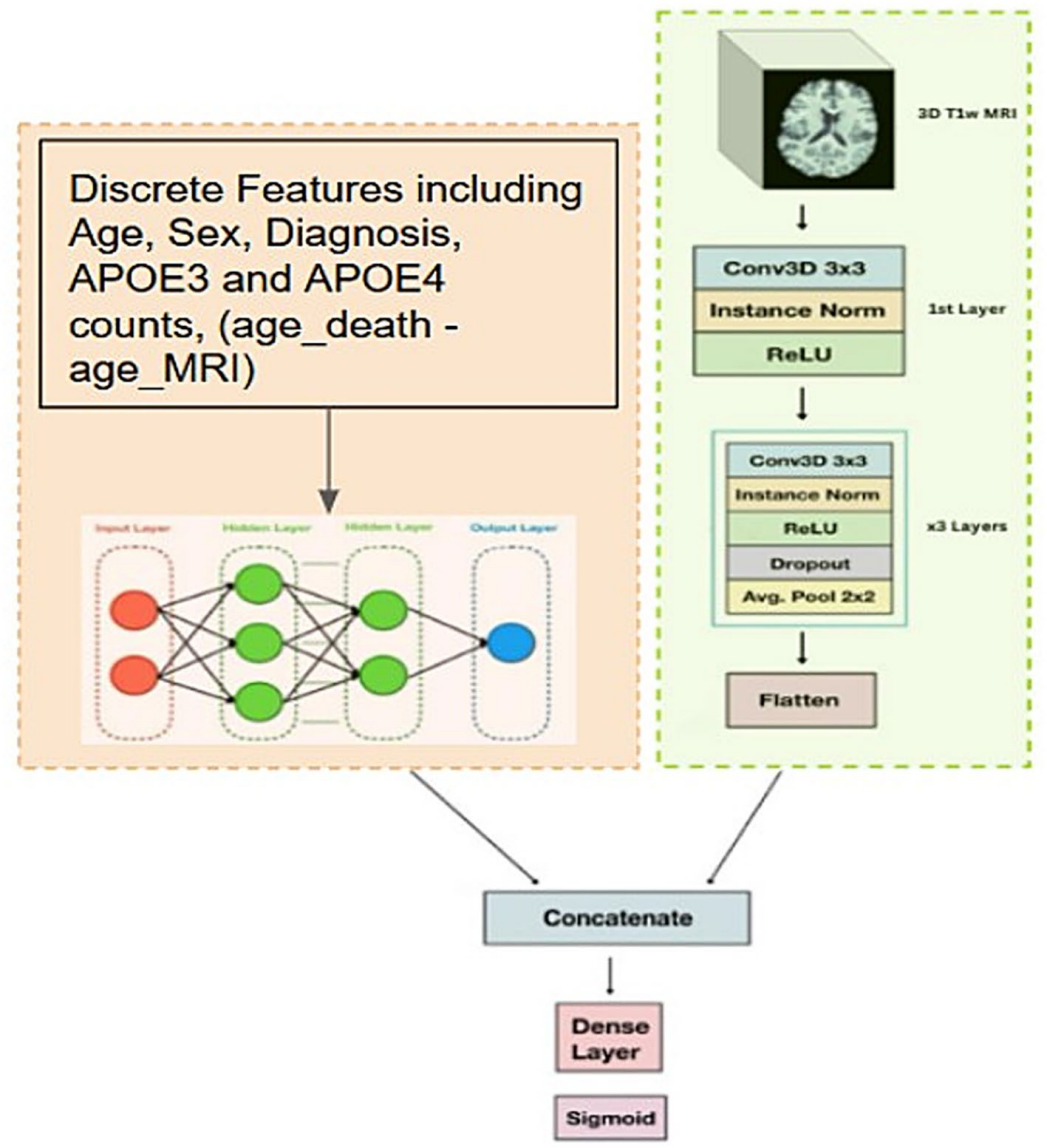
Hybrid 3D CNN Model Architecture. The Y-shaped design merges discrete features in tabular format (left) with entire 3D images (right), which are handled by a 3D CNN.

We designed a hybrid deep learning model (Figure 5) with a ‘Y-shaped’ (29) structure that combines a 3D convolutional neural network (CNN) and a parallel artificial neural network (ANN) to jointly analyze imaging and non-imaging data for classifying neuropathology. The CNN branch processes volumetric T1-weighted MRI scans through a cascade of convolutional layers with progressively larger filter sizes (32, 64, 128, 256), each followed by instance normalization and max pooling layers. The last convolutional block uses average pooling to capture global spatial features while reducing the dimensionality of the resulting feature maps. Simultaneously, the ANN branch takes normalized tabular inputs such as age, sex, diagnosis, time intervals relative to imaging and autopsy, and APOE genotype counts. These inputs pass through three fully connected layers with sizes 1,024, 512, and 64, each incorporating instance normalization and ReLU activation. Categorical data undergo one-hot encoding before entering the ANN. Outputs from both the CNN and ANN streams are concatenated and fed into subsequent dense layers to deliver the final prediction. The entire model is trained end-to-end using the Adam optimizer with a learning rate of 0.001 and batch size of two, for a maximum of 100 epochs, employing early stopping based on validation loss. Performance is measured via balanced accuracy and F1 score.

We applied this hybrid framework across several classification scenarios. Initially, individual binary classifiers were trained for each neuropathology class: Aβ, tau, CAA, TDP-43, HPSC, and DLB. This was then expanded to a multilabel classification approach, enabling simultaneous prediction of multiple co-occurring pathologies by applying sigmoid activations on the output layer, thereby producing independent probabilistic scores for each pathology. This design captures the non-exclusive nature of neuropathological overlap. Optimization in this multilabel setting was guided by a binary cross-entropy loss summed across all target labels. Since label availability varied—with more samples available for amyloid-*β*, Lewy body pathology, and HPSC compared to CAA and TDP-43—we also conducted a focused multilabel experiment restricted to these three more frequently annotated pathologies to leverage a larger training subset and potentially improve model generalization. Further, we incorporated tau pathology using Braak staging (50, 51) to create a six-label multilabel classification. For tau, two additional analyses were performed: one treating Braak stage as a seven-class categorical variable (0–6), and another as a binary classification distinguishing low (≤3) versus high (>3) tau burden. xAI methods were not implemented on this hybrid model due to the inherent difficulty in accurately attributing the influence of input features, as the integrated structure combines distinct processing streams through the CNN and ANN arms, making it challenging to disentangle and assign clear, separate contributions to predictions from each component.

For the 3D CNN and hybrid CNN, the random seed used for all experiments was fixed at 42 for NumPy, PyTorch, and Python random number generators. Data splits were performed at the subject level prior to any model training, with an 80:10:10 train/validation/test ratio, stratified by neuropathological label to preserve class balance across splits. Subjects with multiple MRI scans were assigned exclusively to a single split, and no subject appeared in more than one subset. For AutoGluon experiments, the same data splits were used to ensure comparability across models. The code implementing all models and experiments will be made publicly available on GitHub upon acceptance of the manuscript, including all preprocessing scripts, model architectures, training configurations, and evaluation procedures, to facilitate full reproduction of the reported results.

### 3.6 Transformer-based architecture

To assess whether transformer-based models offer advantages for neuropathology prediction in autopsy-confirmed, moderate-sized datasets, we evaluated two representative vision transformer architectures: a standard Vision Transformer (ViT-B/16) (52) and a compact transformer designed for neuroimaging, MINiT (53). These models were selected to span both high-capacity and lightweight transformer designs commonly proposed for medical imaging. Both architectures operate by partitioning the 3D T1-weighted MRI volume into non-overlapping volumetric patches, which are linearly projected into a latent embedding space and augmented with learnable positional encodings. A classification token is appended to the resulting token sequence, which is then processed by stacked transformer encoder blocks consisting of multi-head self-attention and feed-forward sublayers. The final prediction is derived from the embedding associated with the classification token, representing a global summary of the input volume. The ViT-B/16 model is a high-capacity transformer adapted for volumetric neuroimaging by extending patch tokenization to 3D, enabling modeling of long-range spatial dependencies across the entire brain. In contrast, MINiT is a compact transformer variant optimized for neuroimaging, with a reduced embedding dimension and fewer encoder layers to improve computational efficiency while retaining attention-based contextual modeling. Both transformer models were trained from a random initialization using the same data splits, preprocessing pipeline, and evaluation metrics as the CNN-based models to ensure a controlled comparison. Hyperparameters for transformer training were selected via randomized search over learning rate, weight decay, number of attention heads, and encoder depth, with early stopping based on validation performance.

## 4 Results

To evaluate the incremental contribution of MRI beyond clinical and genetic covariates, we compared covariate-only, imaging-only, and multimodal models across all neuropathological targets. Covariate-only models achieved moderate to strong performance for several outcomes, confirming the predictive value of demographic and genetic risk factors. However, incorporating T1-weighted MRI consistently improved performance for key pathologies such as amyloid-*β*, tau, and cerebral amyloid angiopathy, reflected by higher balanced accuracy and F1 scores in multimodal models. Imaging-only models further showed that structural MRI contains independent discriminative signals, although optimal performance was achieved when imaging was combined with non-imaging covariates. These findings indicate that MRI provides complementary information rather than redundant signals and contributes meaningfully to multimodal neuropathology prediction.

**Figure 6:**
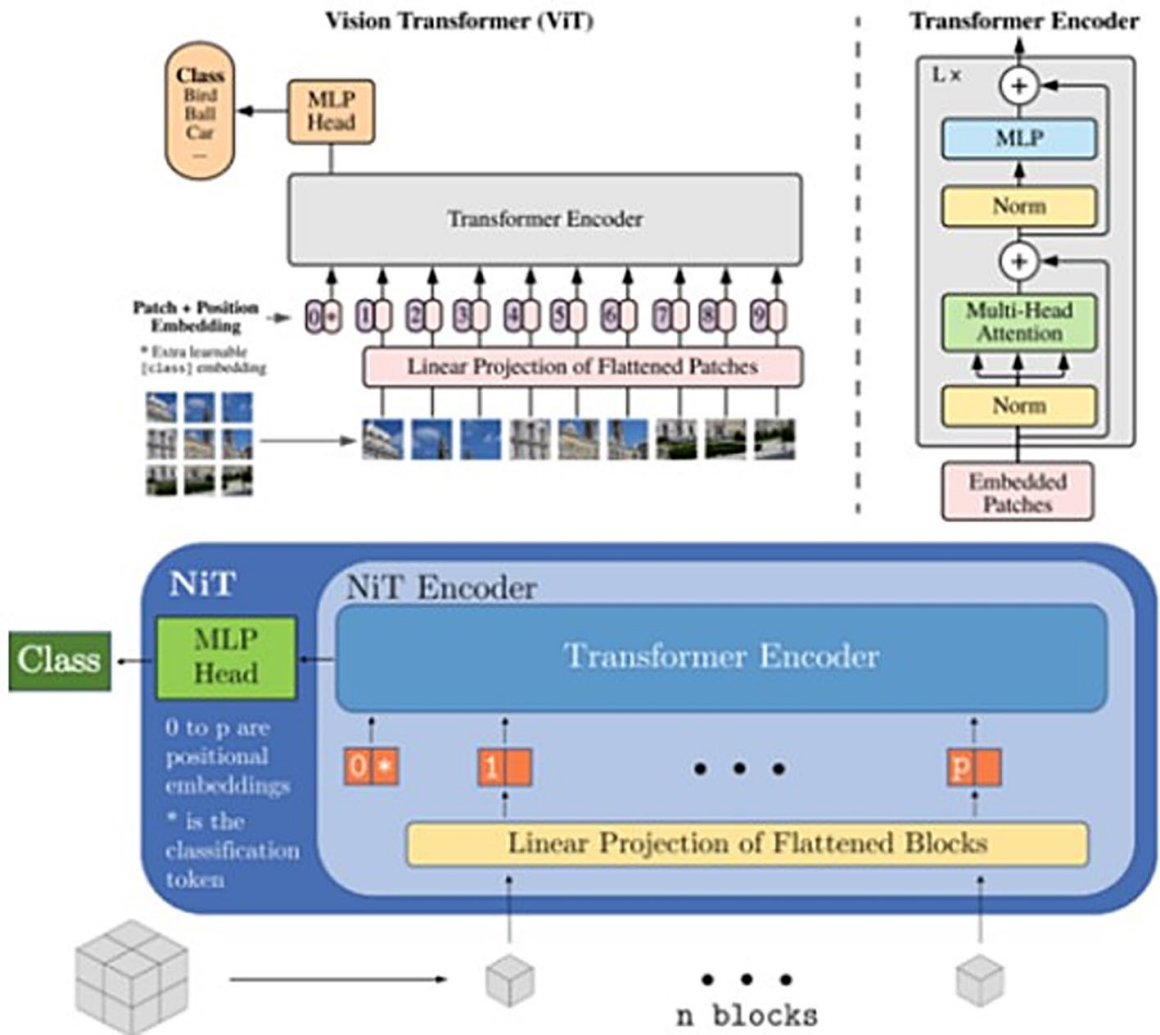
Overview of volumetric vision transformer models used for neuropathology prediction. Both ViT-B/16 and MINiT operate by partitioning 3D T1-weighted MRI volumes into non-overlapping volumetric patches, which are embedded as tokens and processed using stacked self-attention encoder layers. A classification token aggregates global information for downstream prediction. ViT-B/16 is a high-capacity transformer adapted for 3D neuroimaging, whereas MINiT is a compact, neuroimaging-oriented transformer with reduced embedding dimensionality and fewer encoder layers. Although they can model long-range spatial dependencies, both architectures exhibited limited performance in moderate-sized autopsy-confirmed datasets. Architectural diagrams are reproduced from (52, 53).

Across all models, our framework successfully predicted multiple dementia-related neuropathologies from MRI and multimodal data. The best overall performance was achieved by the hybrid 3D CNN model, which integrated T1-weighted MRI with clinical and genetic covariates, reaching balanced accuracies up to 0.76 and F1-scores up to 0.83 for key targets such as amyloid-*β*, tau, and CAA. The 3D CNN alone performed well for amyloid (Bal. Acc. = 0.72, F1 = 0.87) and TDP-43 (Bal. Acc. = 0.79), while AutoGluon models incorporating imaging features also showed strong results, particularly for amyloid and Braak tau staging. Overall, performance improved consistently when imaging was combined with demographic and genetic covariates, confirming the added value of multimodal integration (see Figure 6).

**Figure 7:**
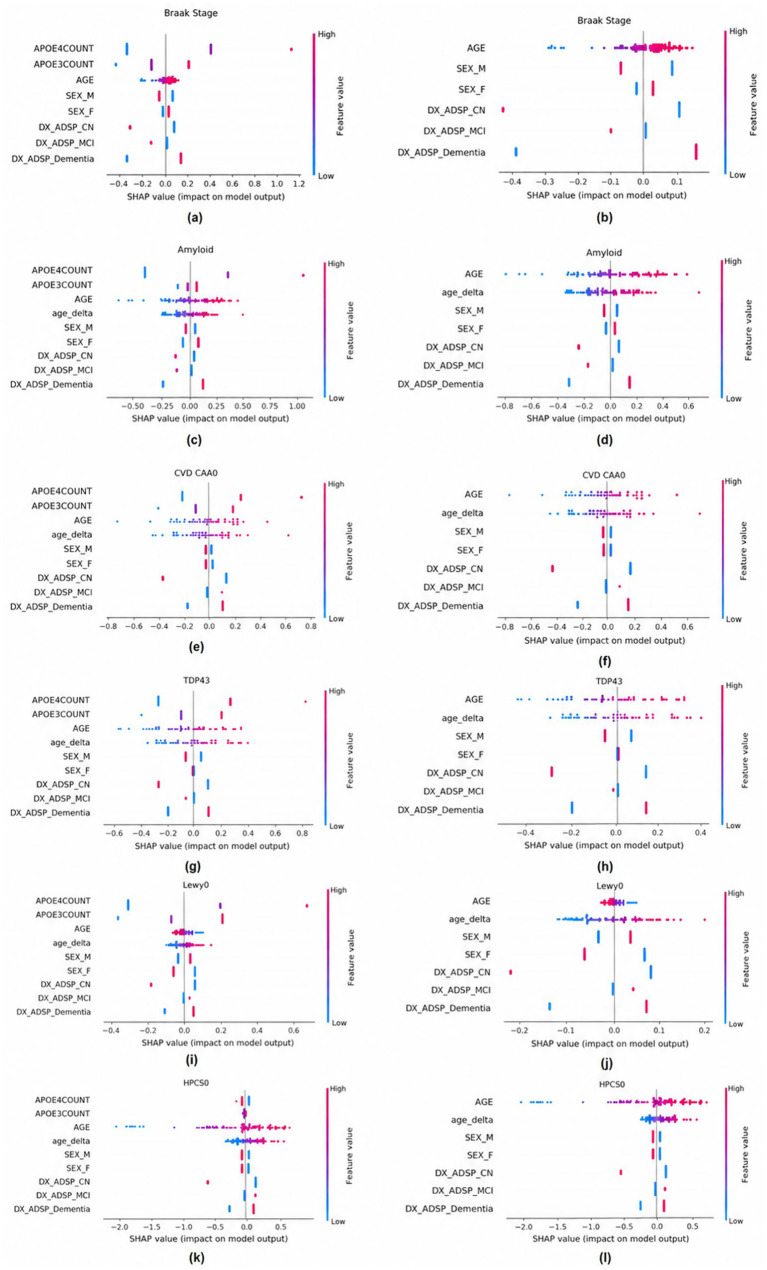
Shapley value plots for **(a,b)** Tau Braak Stages; **(c,d)** Amyloid; **(e,f)** cerebral amyloid angiopathy (CAA); **(g,h)** transactive response DNA-binding protein 43 (TDP-43). **(i,j)** presence of Lewy Body (DLB). **(k,l)** hippocampal sclerosis (HPSC). The plots display the overall importance of each feature in the model, with colors indicating the feature value––red means high and blue means low. The horizontal spread of the data points shows how much the feature impacts the model’s predictions. In the plots, continuous variables (e.g., age, time interval between imaging and autopsy) display a horizontal spread because each subject contributes a distinct numerical value, allowing the SHAP values to vary smoothly across their range. In contrast, categorical covariates (e.g., sex or diagnosis) have discrete levels, so their SHAP values cluster into vertical bands with minimal horizontal dispersion.

For the Random Forest algorithm, our results are summarized in Table 2. For most of the neuropathologies apart from HPCS and DLB, performance improved when adding information on APOE alleles in the covariates used for training. The Shapley Plots highlighted age at imaging, time intervals relative to imaging and autopsy and APOE4 allele counts as the most influential features for predicting the neuropathologies (Figure 7). These results show the models’ reliance on clinically meaningful predictors and are consistent with established neuropathological risk factors.

**Table 2:** Random forest experiment results.

Covariates used	Amyloid		CAA		TDP-43		DLB		HPSC		Braak	
	Bal Acc.	F1	Bal Acc.	F1	Bal Acc.	F1	Bal Acc.	F1	Bal Acc.	F1	Bal Acc.	F1
Covariates used: APOE4COUNT, APOE3COUNT, AGE, SEX, DX_ADSP, age_delta	**0.674**	**0.626**	**0.783**	**0.693**	**0.786**	**0.763**	0.556	0.537	0.647	0.482	**0.717**	**0.717**
Covariates used: AGE, SEX, DX_ADSP, age_delta	0.581	0.573	0.653	0.590	0.766	0.755	**0.627**	**0.571**	**0.660**	**0.497**	0.657	0.660

Bal Acc. stands for Balanced Accuracy and F1 stands for F1 Score. Best scores are in Bold.

For the AutoGluon experiments, Table 3 summarizes the classification performance of various models. Using only covariates such as APOE genotype counts, sex, diagnosis, and age delta, the models achieved moderate performance, with the best results observed for TDP-43 (Bal. Acc. = 0.862, F1 = 0.870) and HPSC (Bal. Acc. = 0.705, F1 = 0.553). Incorporating T1w image features in the multimodal single-slice model substantially improved classification accuracy for several pathologies. Specifically, the coronal slice configuration achieved the highest overall performance, with Amyloid classification reaching Bal. Acc. = 0.755 and F1 = 0.923, while the binarized version of Braak staging also benefited (Bal Acc. = 0.739 and F1 = 0.838, respectively). The all-slices multimodal model did not yield further gains and, in some cases, reduced performance compared to the single-slice configuration, suggesting that a representative slice with covariates captures sufficient discriminatory information for most neuropathological outcomes.

**Table 3:** AutoGluon experiment results.

Input	Amyloid		CAA		TDP-43		DLB		HPSC		Braak	
	Bal Acc.	F1	Bal Acc.	F1	Bal Acc.	F1	Bal Acc.	F1	Bal Acc.	F1	Bal Acc.	F1
Covariates only model												
Covariates used: APOE4COUNT, APOE3COUNT, AGE, SEX, DX_ADSP, age_delta	0.642	0.659	0.794	0.746	**0.862**	**0.870**	0.624	0.628	**0.705**	**0.553**	**0.739**	0.747
Covariates used: AGE, SEX, DX_ADSP, age_delta	0.689	0.669	**0.842**	**0.864**	0.842	0.864	**0.679**	**0.686**	0.681	0.688	0.712	0.718
Multimodal Single Slice + Covariates (APOE4COUNT, APOE3COUNT, AGE, SEX, DX_ADSP, age_delta) Model												
Axial Slice	**0.755**	**0.923**	0.609	0.400	0.607	0.353	0.635	0.575	0.562	0.222	**0.739**	**0.838**
Coronal Slice	**0.755**	**0.923**	0.609	0.400	0.543	0.444	0.635	0.575	0.562	0.222	**0.739**	**0.838**
Multimodal all slices + Covariates model												
Covariates used: APOE4COUNT, APOE3COUNT, AGE, SEX, DX_ADSP, age_delta	0.743	0.684	0.709	0.695	0.626	0.618	0.586	0.582	0.484	0.456	0.736	0.742
Covariates used: AGE, SEX, DX_ADSP, age_delta	0.584	0.583	0.554	0.556	0.791	0.809	0.614	0.617	0.692	0.461	0.692	0.697

Bal Acc. stands for Balanced Accuracy and F1 stands for F1 Score. Best scores are in Bold.

For the 3D CNN, which uses only the images as input, Table 4 summarizes the results. The model demonstrated varying levels of discriminative ability across different neuropathological targets. The best performance was achieved for Amyloid classification, with a Balanced Accuracy of 0.721 and an F1-score of 0.867, followed by TDP-43 (Bal. Acc. = 0.792, F1 = 0.684), indicating that structural MRI contains discernible features related to these pathologies. In contrast, performance for CAA, DLB, and HPCS was relatively lower, particularly for HPCS (F1 = 0.187), suggesting limited sensitivity of T1w-derived morphometric features for these conditions. Overall, while the 3D CNN captured relevant structural patterns for certain neuropathologies, its standalone performance underscores the need for incorporating additional modalities or covariates to improve classification robustness and generalizability.

**Table 4:** 3D CNN experiment results.

Amyloid		CAA		TDP-43		DLB		HPSC		Braak	
Bal Acc.	F1	Bal Acc.	F1	Bal Acc.	F1	Bal Acc.	F1	Bal Acc.	F1	Bal Acc.	F1
Image Only Model (Input–3D T1w)											
0.721	0.867	0.530	0.392	0.792	0.684	0.541	0.426	0.556	0.187	0.638	0.687

Bal Acc. stands for Balanced Accuracy and F1 stands for F1 Score.

**Figure 8:**
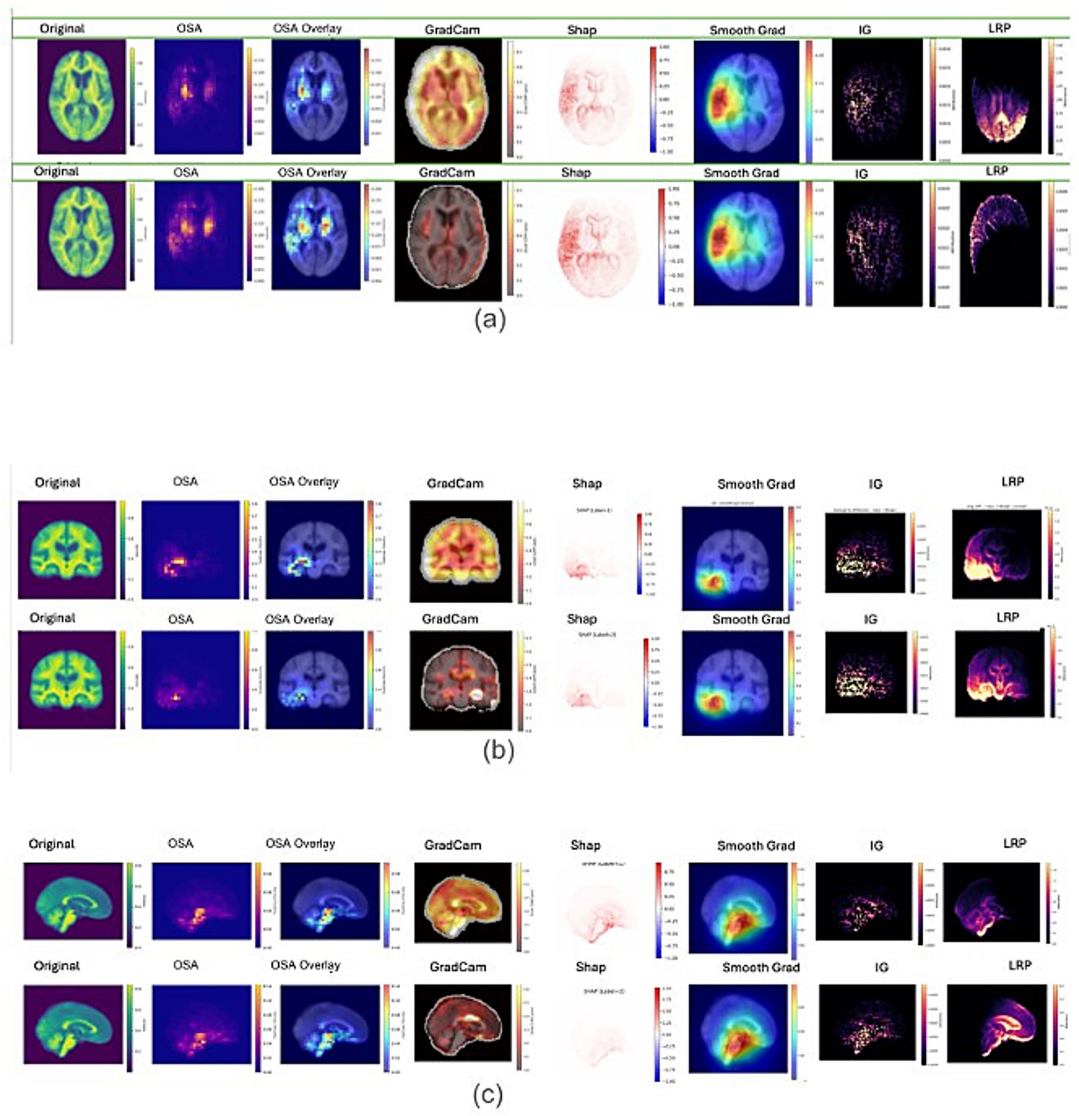
**(a–c)**––Results for the xAI models for Tau Braak Stage for the 3D CNN Architecture in **(a)**––axial, **(b)** coronal, and **(c)**––sagittal views. For each figure, the top row is for average positive class prediction for Tau Braak Stage, and the bottom row is for the average negative class prediction for Tau Braak Stage.

**Figure 9:**
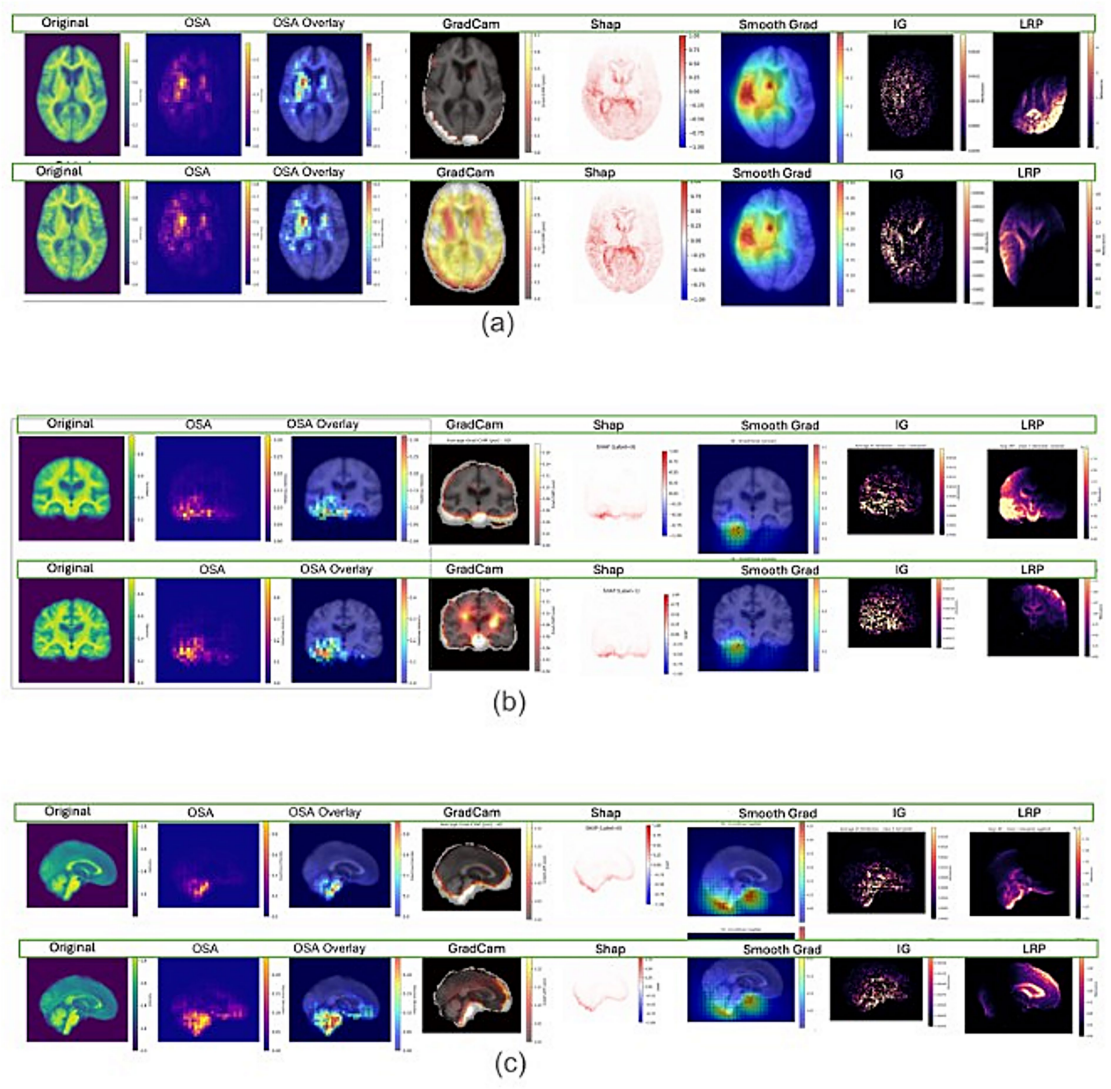
**(a–c)**––Results for the xAI models for Amyloid for the 3D CNN Architecture in **(a)**––axial, **(b)** coronal, and **(c)**––sagittal views. For each figure, the top row is for average positive class prediction forAmyloid, and the bottom row is for the average negative class prediction for Amyloid.

**Figure 10:**
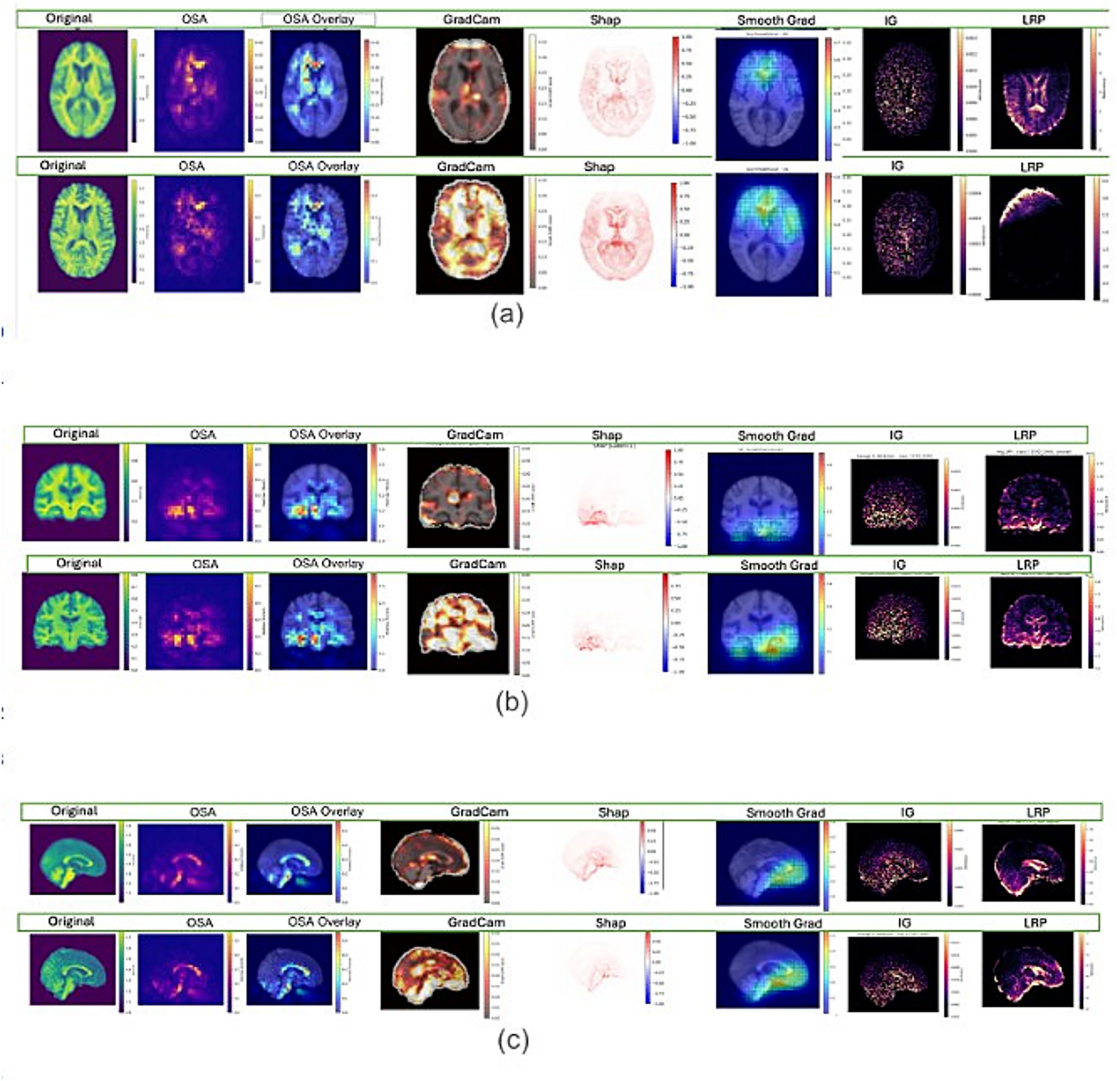
**(a–c)**––Results for the xAI models for cerebral amyloid angiopathy (CAA) for the 3D CNN Architecture in **(a)**––axial, **(b)** coronal and **(c)**––sagittal views. For each figure, the top row is for average positive class prediction for CAA, and the bottom row is for the average negative class prediction for CAA.

**Figure 11:**
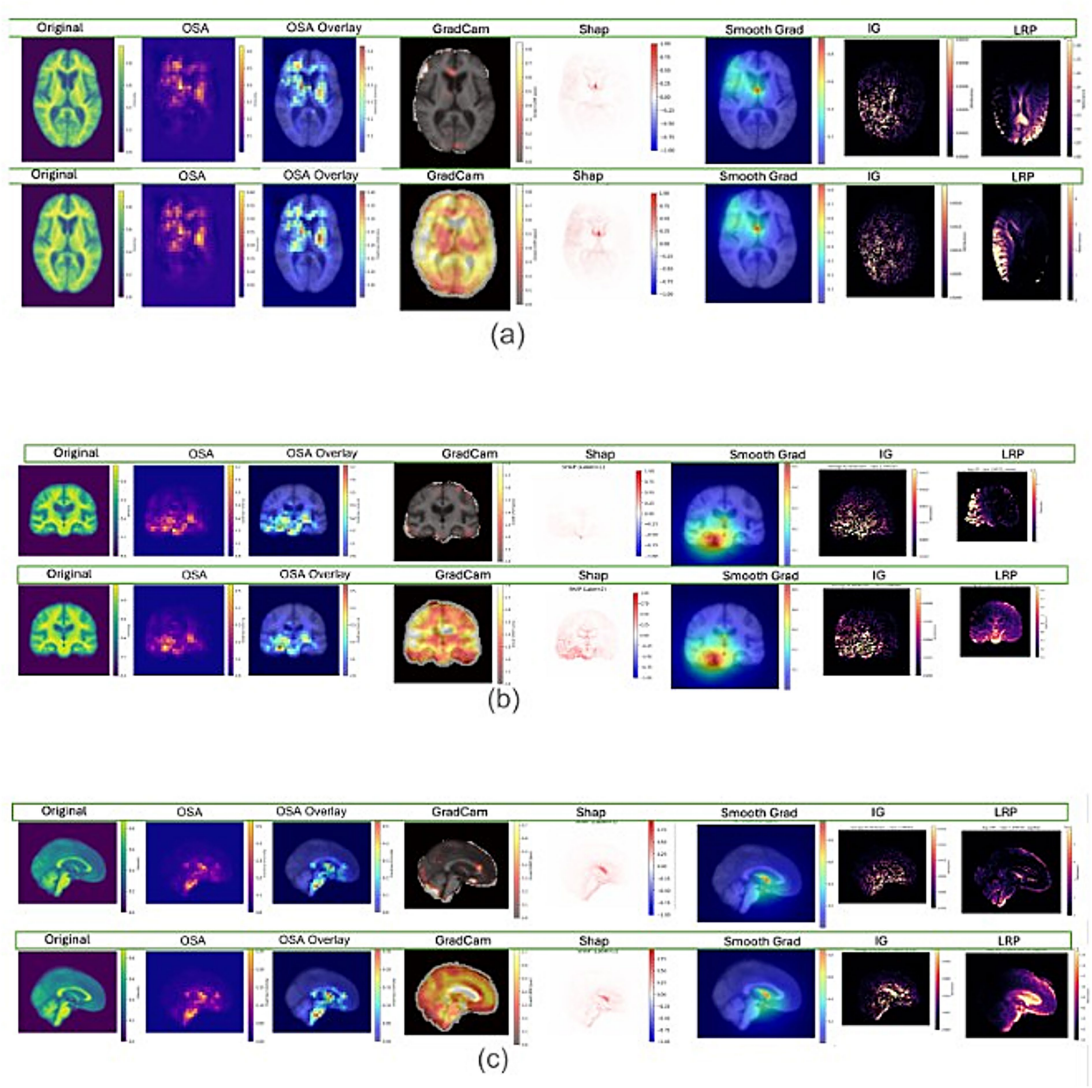
**(a–c)**––Results for the xAI models for hippocampal sclerosis (HPSC) for the 3D CNN Architecture in **(a)**––axial, **(b)** coronal and **(c)**––sagittal views. For each figure, the top row is for average positive class prediction for HPSC, and the bottom row is for the average negative class prediction for HPSC.

**Figure 12:**
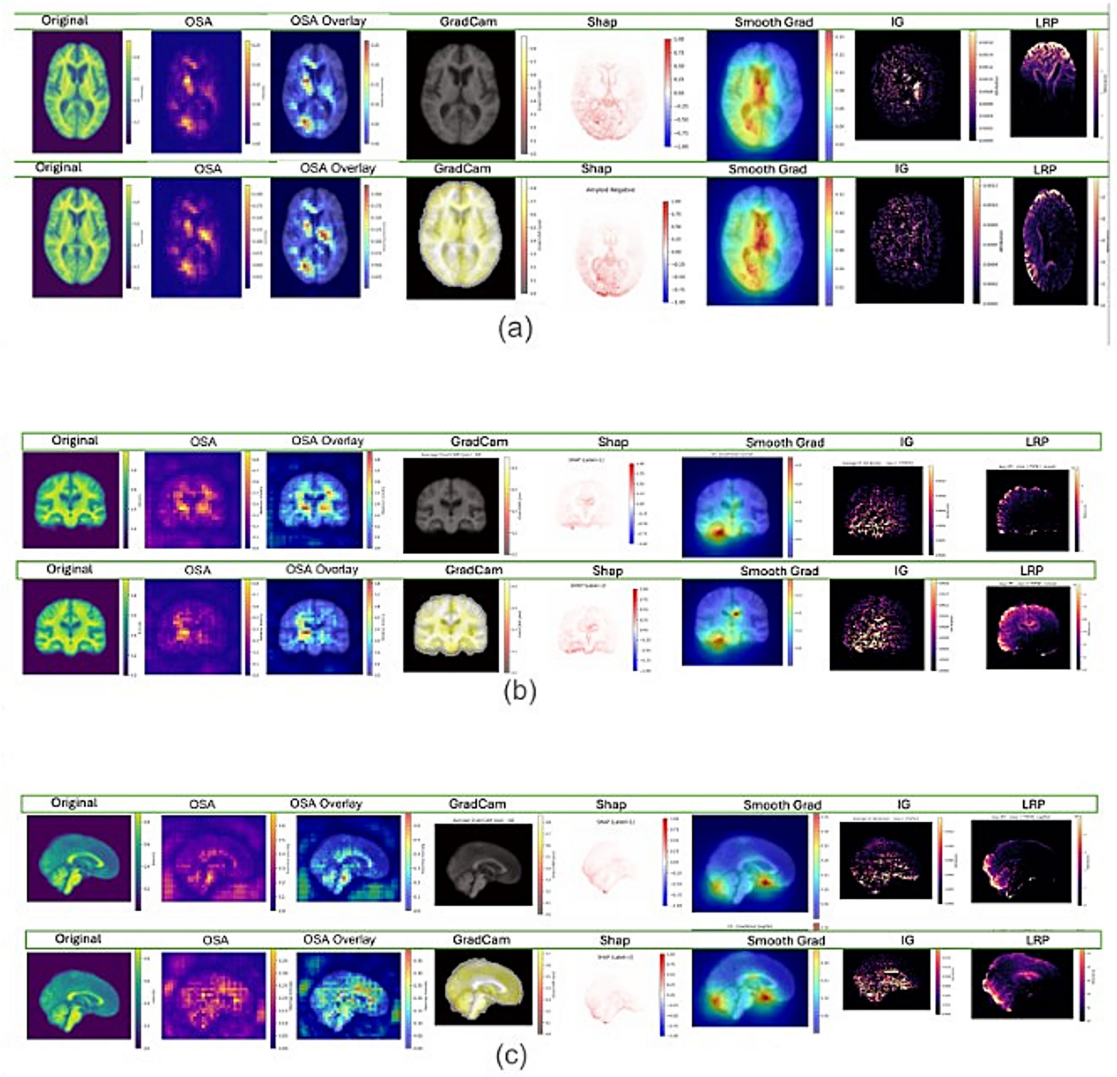
**(a–c)**––Results for the xAI models for transactive response DNA-binding protein 43 (TDP-43) for the 3D CNN Architecture in **(a)**––axial, **(b)** coronal and **(c)**––sagittal views. For each figure, the top row is for average positive class prediction for TDP-43, and the bottom row is for the average negative class prediction for TDP-43.

**Figure 13:**
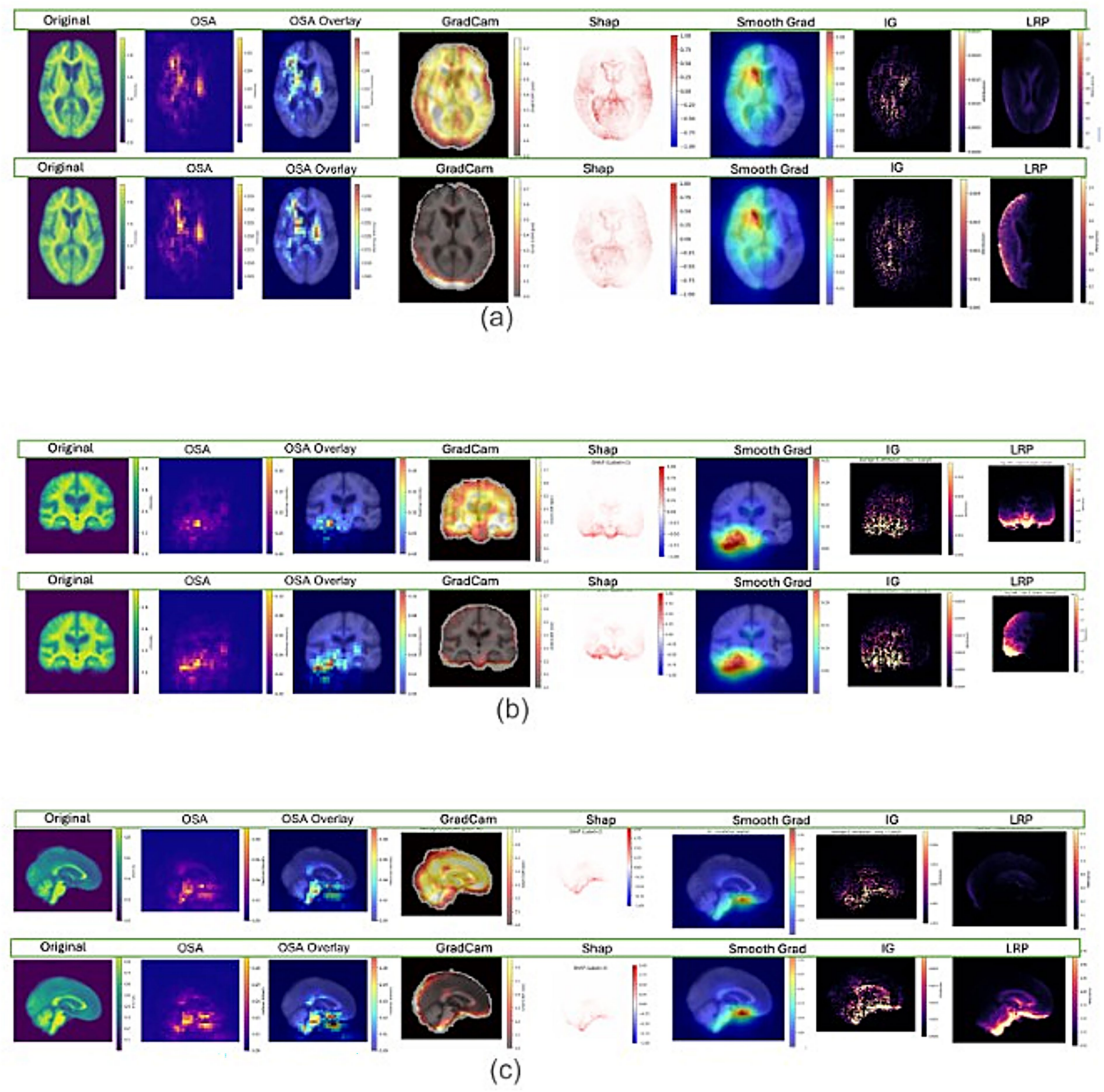
**(a–c)**––Results for the xAI models for Lewy Body Pathology (LB) for the 3D CNN Architecture in **(a)**––axial, **(b)** coronal and **(c)**––sagittal views. For each figure, the top row is for average positive class prediction for LB, and the bottom row is for the average negative class prediction for LB.

Figures 8–13 show the results of the various xAI models for each Neuropath in three views––axial, coronal and sagittal for the respective middle slices. The first column for each figure is the actual original slice, the second column is the OSA implementation, and the third column is the OSA map overlaid over the original image. The next columns are GradCAM, Shapley Map, Smooth Grad, Integrated Gradients Map and Layer-wise Relevance Propagation, respectively. For each figure, the top row is for average positive class prediction for each neuropath, and the bottom row is for the average negative class prediction for each neuropath. For most neuropaths, across xAI models, consistent regions were picked up to be contributing the most important features for classification performance.

**Figure 14:**
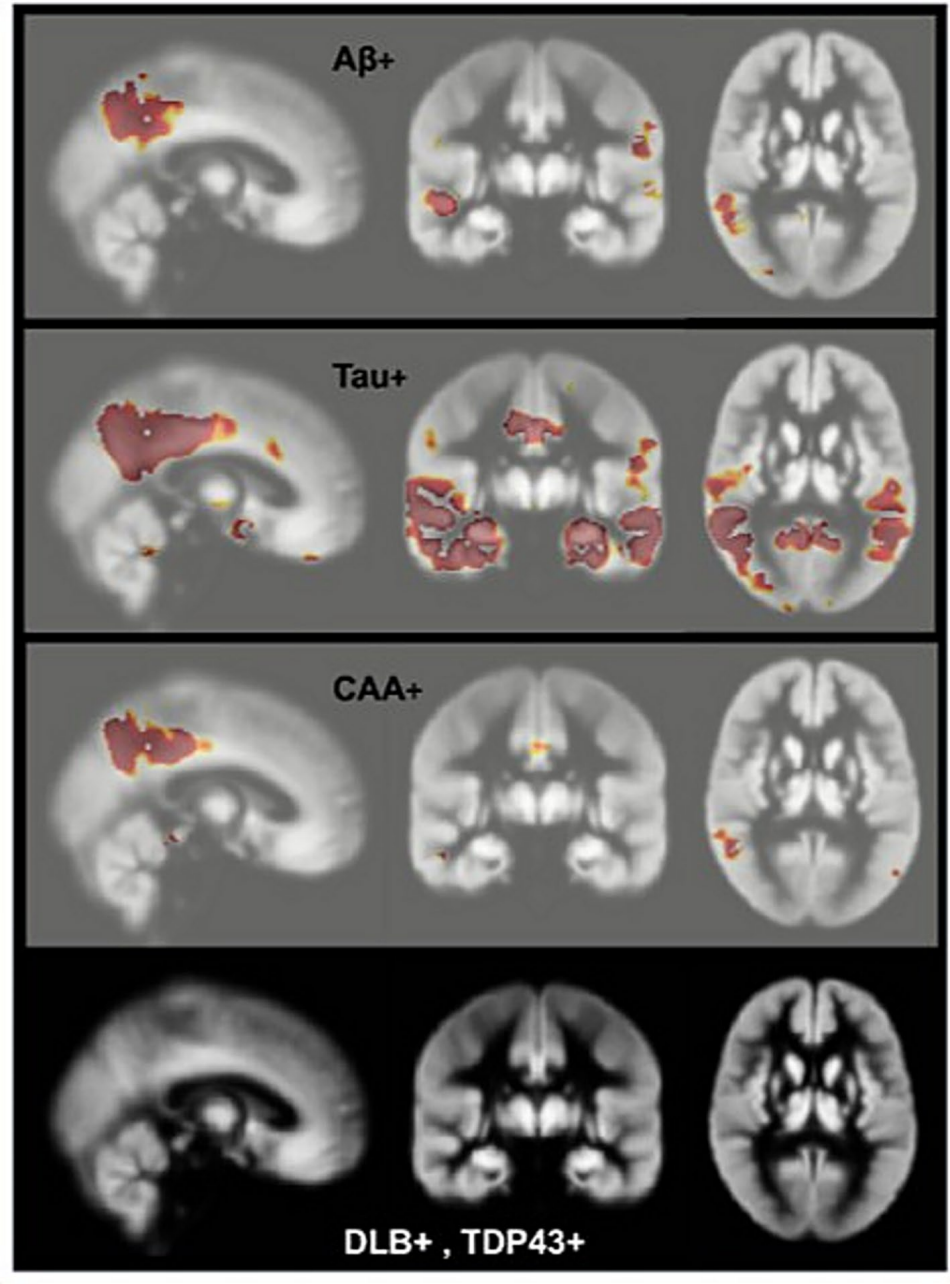
Visualization of VBM significant regions overlaid on standard brain template. Interestingly, the posterior cingulate region (upper left panel), which is one of the earliest to be affected in AD, shows atrophy that is associated with both amyloid positivity and the presence of CAA (left panel of the CAA results). By far the most profound association is between tau positivity and atrophy of the temporal lobes, and to some extent with atrophy of the same posterior cingulate region associated with amyloid positivity and CAA. It is well known that tau pathology is linked with atrophy, especially in the medial temporal regions that show earliest and greatest tau burden.

Voxel-based morphometry (VBM) analyses revealed significant associations between regional gray matter volume (GMV) alterations and the presence of neuropathological markers for Amyloid-beta, Tau, and Cerebral Amyloid Angiopathy (Figure 14). Statistical significance was established after correction for multiple comparisons using the False Discovery Rate (FDR) method at a threshold of q = 0.05, highlighting robust neuroanatomical correlates of these pathologies (Table 5). Significant GMV differences were observed in individuals positive for Aβ, Tau, and CAA, with FDR-corrected *p*-values of 0.0039, 0.0161, and 0.0026, respectively. This indicates that these pathologies are reliably associated with altered brain structure across the studied age span (38.5–100.1 years). No statistically significant alteration in GMV was detected for TDP43 + and LB + groups after FDR correction, suggesting either less pronounced volumetric effects or insufficient sample size in these subgroups. Visualization of the significant regions (overlaid on standard brain template) shows consistent spatial patterns for Aβ+, Tau+, and CAA+, helping to localize areas of GMV reduction most affected by these neuropathologies.

**Table 5:** VBM experiment results.

Neuropath	Total N	NPath(+) N	Age Range (years)	*P*-val (FDR-corrected)
Amyloid	513	439	38.5–100.1	0.003*
Braak Tau	501	350	38.5–100.1	0.016*
TDP-43	241	76	38.5–97.4	null
CAA	512	336	38.5–100.1	0.003*
LB	512	206	38.5–100.1	null

*Indicates statistically significant FDR-corrected *P* < 0.05. Bold values denote statistical significance. NPath(+) N indicates the number of subjects who were neuropathologically positive, excluding the control group.

### 4.1 Congruence of xAI salience maps and VBM

Comparing these results to the xAI models show considerable overlap between the VBM maps of pathology-related atrophy and the salience maps that identify brain regions that contribute to classifying neuropaths for the deep learning models. Three general principles are evident: first, with some exceptions, there is not a strong agreement in the regions used by the different deep learning methods, an observation also noted by (44) in their comparison of xAI methods. In the case of Tau pathology (Figure 8), there is a strong and coherent signal in the temporal lobes, seen in both the SmoothGrad and LRP interpretability maps, which is the same region strongly implicated in the VBM results. This is also the region where tau pathology first accumulates, and where atrophy is greatest in classical AD (54, 55). In addition to these results for tau, the xAI maps for hippocampal sclerosis do indeed selectively emphasise the hippocampus, among other regions, which makes sense the HPSC is the most localized pathology and by definition it is expected to be localized to the hippocampus. Beyond this agreement, the second general trend is that there are several strong signals in regions that would not be expected *a priori*, such as the brain stem regions, where atrophy is most strongly linked with mortality but is not prominent or characteristic in early AD. Finally, many of the salience maps are strongly lateralized, which is not expected as none of the pathologies tend to be lateralized overall, even though they may be in some individual cases. This asymmetry is clearly evident in all the maps for Lewy bodies, and is evident in all the SmoothGrad maps for every pathology. It is not clear why this effect emerges, as the features that are diagnostic might be expected to be evident to some extent in both hemispheres. The xAI methods based on occlusion assess the value of a feature when the contralateral feature is still present in the image, so it could be that the approach is identifying an asymmetric feature, as brain asymmetry increases in normal aging and to an even greater degree with dementia.

### 4.2 Classification performance of the hybrid CNN

Independent classifiers were developed utilizing the hybrid CNN architecture, which consistently surpassed AutoGluon in classifying nearly all pathology types (Table 6). For example, the balanced accuracy and F1 score for TDP-43 reached 0.719 and 0.690, respectively, whereas for CAA, these metrics were 0.763 and 0.711. This enhanced performance is likely attributable to the model’s capacity to integrate comprehensive three-dimensional spatial information along with additional demographic and clinical inputs. To further explore multi-pathology classification, the hybrid CNN was assessed under multilabel scenarios. Initially, analysis was confined to the three most commonly evaluated pathologies; amyloid, Lewy body pathology, and HPSC; in order to maximize sample size. Although amyloid detection maintained strong accuracy, there was a notable decline in F1 score for Lewy bodies (0.488) and HPSC (0.208), potentially reflecting lower prevalence or more subtle imaging signatures for these conditions. Expanding the multilabel classification to include CAA and TDP-43 demonstrated high F1 scores for amyloid and CAA; however, the ability to correctly classify Lewy bodies and TDP-43 was further diminished, possibly due to increased label sparsity and overlapping imaging characteristics within the training set. In the most extensive model, tau pathology (using Braak staging) was also incorporated. The classifier continued to perform strongly on amyloid, tau, and CAA, indicating that the integrated multimodal system can reliably handle multiple concurrent pathologies in a joint training context. Tau categorization was also separately analyzed as both a binary and multiclass problem; the binary approach consistently outperformed the multiclass (seven-category) setup. Specifically, the binary classifier achieved F1 scores of 0.831 individually and 0.818 in the comprehensive multilabel context, while performance in the multiclass framework was reduced (F1 score of 0.463), likely due to challenges differentiating contiguous Braak stages on MRI and imbalanced sample distributions. These findings further illustrate the trade-off between the completeness of available pathology labels and the range of model coverage.

**Table 6:** Hybrid CNN experiment results.

Model	Amyloid		CAA		TDP-43		DLB		HPSC		Braak 2 Class		Braak Multi Class	
	Bal Acc.	F1	Bal Acc.	F1	Bal Acc.	F1	Bal Acc.	F1	Bal Acc.	F1	Bal Acc.	F1	Bal Acc.	F1
Hybrid CNN Individual Classifiers	0.744	0.695	**0.763**	0.711	0.719	**0.609**	0.671	**0.683**	0.669	0.255	0.758	**0.831**	0.735	0.463
Hybrid CNN MultiLabel Classifier for 3 Neuropaths	**0.850**	**0.965**	-	-	-	-	0.652	0.488	0.679	0.208	-	-	-	-
Hybrid CNN MultiLabel Classifier for all Neuropaths except Tau	0.740	0.901	0.710	0.733	**0.735**	0.520	**0.690**	0.603	**0.732**	0.208	-	-	-	-
Hybrid CNN MultiLabel Classifier for all Neuropaths and Tau	0.781	0.927	0.681	**0.810**	0.713	0.400	0.669	0.591	0.720	**0.456**	**0.763**	0.818	-	-

Bal Acc. stands for Balanced Accuracy and F1 stands for F1 Score. Best scores are in Bold.

To assess the impact of temporal separation between MRI acquisition and autopsy on model performance, we performed a stratified evaluation of the hybrid CNN classifiers based on the interval between imaging and death (age_delta). Age_delta ranged from 0.10 to 15.0 years in the test set, with a median of 4.30 years. The test cohort was therefore divided into two groups: shorter interval (≤4.30 years; N = 55) and longer interval (>4.30 years; N = 54). Classification performance for each neuropathology was evaluated separately within these two strata using balanced accuracy and F1 score, without retraining the models. Results are summarized in Table 7. For most neuropathologies, performance was comparable across interval groups or modestly higher in the longer-interval subgroup. Amyloid-*β* prediction remained highly stable across both strata (balanced accuracy ≈ 0.80), while CAA, TDP-43, and hippocampal sclerosis demonstrated improved balanced accuracy and F1 score in the longer-interval group. Lewy body pathology showed a moderate decrease in performance at longer intervals, whereas binary tau classification exhibited minimal differences across strata. Overall, these results show that predictive performance does not systematically decline with increasing MRI-to-autopsy interval within the observed range, suggesting temporal robustness of the learned imaging and multimodal signatures.

**Table 7:** Effect of MRI-to-autopsy interval on hybrid CNN classification performance.

HybridCNN	Amyloid		CAA		TDP-43		DLB		HPSC		Braak	
Age delta group	Bal Acc.	F1	Bal Acc.	F1	Bal Acc.	F1	Bal Acc.	F1	Bal Acc.	F1	Bal Acc.	F1
**≤4.30**	0.799	0.829	0.702	0.642	0.619	0.222	0.704	0.679	0.702	0.476	0.784	0.841
**>4.30**	0.808	0.943	0.747	0.706	0.683	0.500	0.645	0.542	0.793	0.600	0.742	0.848

Age_delta denotes the interval between MRI acquisition and death. Models were trained once on the full training set and evaluated on stratified test subsets without retraining.

### 4.3 Transformer-based model performance

Transformer-based models trained from scratch exhibited substantially weaker and less stable performance compared to CNN-based architectures across nearly all neuropathological targets (Table 8). Both ViT-B/16 and MINiT performed close to chance level for several pathologies, with particularly limited discriminative ability for amyloid-*β*, cerebral amyloid angiopathy, and hippocampal sclerosis. While isolated metrics for specific targets appeared competitive, these effects were not consistent and did not generalize across pathologies. Notably, the compact MINiT architecture was not more robust than the larger ViT-B/16 model, suggesting that reduced parameter count alone is insufficient to overcome the data inefficiency of attention-based architectures in this setting. In contrast, CNN-based models consistently achieved higher balanced accuracy and F1 scores, particularly for pathologies with known structural correlates on T1-weighted brain MRI. These findings indicate that, when trained on moderate-sized, autopsy-confirmed datasets without large-scale pretraining, transformer-based volumetric models may struggle to reliably capture disease-relevant neuroanatomical patterns.

**Table 8:** Transformer experimental results.

Model	Amyloid		CAA		TDP-43		DLB		HPSC		Braak	
	Bal Acc.	F1	Bal Acc.	F1	Bal Acc.	F1	Bal Acc.	F1	Bal Acc.	F1	Bal Acc.	F1
VIT-b/16	0.524	0.524	0.501	0.189	0.501	0.413	0.582	0.519	0.501	0.084	0.501	0.396
MINIT	0.519	0.864	0.529	0.288	0.561	0.302	0.510	0.297	0.494	0.011	0.540	0.729

Bal Acc. stands for Balanced Accuracy and F1 stands for F1 Score.

## 5 Discussion

Our results demonstrate that several key dementia-related neuropathologies can be reliably detected from non-invasive MRI when combined with clinical and genetic information. Using the hybrid 3D CNN model, which integrates volumetric T1-weighted MRI with demographic and genetic covariates, we achieved the strongest overall performance, with balanced accuracies up to 0.76 and F1-scores up to 0.83 for amyloid-*β*, tau, and cerebral amyloid angiopathy (CAA). These findings indicate that structural MRI contains informative signatures of these pathologies that can be amplified through multimodal fusion. While detection of TDP-43 and Lewy body pathology was more challenging, the models still captured meaningful discriminative patterns, underscoring the potential of multimodal deep learning for comprehensive, *in vivo* neuropathological profiling.

Among the machine learning baselines, Random Forest models trained on clinical and genetic covariates with APOE data achieved moderate balanced accuracy across most neuropathologies, with performance gains generally seen when APOE alleles were included. However, these models, while interpretable via SHAP values, were limited as they only included non-imaging data and were not informed by neuroanatomical patterns associated with neuropathology. AutoGluon models, using automated machine learning on covariates alone, achieved a comparable range of performance. Importantly, incorporation of 2D slices from T1w MRI in AutoGluon improved classification metrics especially for amyloid and Braak tau stages, indicating the added value of imaging features even when constrained to 2D representations. The 3D CNN model, trained on volumetric T1-weighted MRI, demonstrated robust performance for amyloid and TDP-43 pathologies, highlighting the discriminative power of 3D structural patterns detectable via deep convolutional architectures. Notably, performance for other pathologies including CAA, Lewy Bodies (LB), and hippocampal sclerosis (HPSC) was lower, possibly reflecting subtler or less well-captured morphometric signatures for these conditions in T1w images alone.

The MRI-to-autopsy interval, which ranged up to 15 years, is an important source of label uncertainty. Neuropathological labels derived from post-mortem assessment reflect the state of pathological burden at death, while MRI was acquired at a variable and sometimes substantial time beforehand. For pathologies with slow, progressive accumulation trajectories such as amyloid-*β* and tau, longer intervals may still allow for the possibility that imaging-detectable structural changes were already present at the time of scanning, consistent with the stable or improved classification performance observed for these pathologies in our interval-stratified analysis. For pathologies with more variable or rapidly evolving trajectories such as TDP-43 and Lewy body pathology, longer intervals may introduce greater label uncertainty, which may partly explain the more variable performance observed for these conditions. Label noise introduced by this temporal gap is a limitation of all studies using autopsy-confirmed labels linked to ante-mortem imaging, and is shared by existing work in this space. Future studies incorporating longitudinal MRI data or modeling the imaging-to-autopsy interval as a continuous covariate within the deep learning framework would help to mitigate this source of uncertainty.

The suite of xAI methods applied to the 3D CNN explained spatial patterns in the brain that most strongly influenced model predictions. Across occlusion sensitivity, Grad-CAM, Shapley values, Smooth Grad, Integrated Gradients, and Layer-wise Relevance Propagation, there was convergence on consistent regional features that aligned with the neuropathological targets. These saliency maps highlighted biologically plausible brain regions implicated in amyloid, tau, and CAA pathology. In contrast, voxel-based morphometry (VBM), while firmly established as a neuroanatomical analytic method, revealed robust gray matter volume differences only for amyloid, tau, and CAA after standard multiple comparison correction. The lack of significant VBM findings for TDP-43 and LB suggests these pathologies may not produce pronounced volumetric alterations detectable by VBM or that sample sizes have limited statistical power. Importantly, the overlap between regions implicated by xAI maps and VBM findings for amyloid, tau, and CAA supports the biological validity of the deep learning model explanations. Yet, the xAI methods offer several advantages over VBM: they capture complex, multivariate, hierarchical features learned directly by the classification model rather than simple volumetric changes; they provide subject-specific, model-driven interpretability that can identify subtle imaging features beyond volumetric differences; and they are integrally linked to predictive performance rather than solely group-level anatomical differences. While there was partial overlap between the xAI-derived salience maps and VBM results, it is important to interpret these findings with caution. Recent work (44) has shown that commonly used xAI techniques often produce non-overlapping and inconsistent saliency maps, even when applied to the same model and dataset, reflecting the non-local and hierarchical nature of feature representations in deep vision networks. As these models rely on distributed features across multiple abstraction levels, voxel-wise feature attribution does not fully capture the mechanisms driving predictions. Consequently, we do not interpret individual-level xAI maps as direct indicators of where pathology resides, but rather as tools to understand, on average, which regions or patterns the models emphasize during decision-making. It should be noted that the voxel-based morphometry analyses were conducted on a subset of participants (N = 512) who met stringent preprocessing and quality-control criteria, using a single scan per subject in line with standard VBM practice. In contrast, the deep learning models were trained and evaluated on a larger dataset that included multiple scans per subject when available, with scan-specific covariates appropriately modeled and subject-level separation enforced across training, validation, and test sets. As a result, comparisons between group-level VBM maps and averaged CNN salience maps are qualitative in nature and should not be interpreted as direct, quantitative correspondence. Instead, these comparisons are intended to provide contextual validation by assessing whether imaging regions emphasized by the deep learning models broadly align with established neuroanatomical patterns. The xAI saliency maps in this study should be interpreted with care. These maps reflect regions whose perturbation or occlusion most strongly influences model output, i.e., they indicate model attention patterns rather than directly localizing pathology within the brain. Several observations in the saliency maps, including unexpected lateralization across multiple pathologies and brainstem emphasis in some conditions, likely reflect methodological properties of the attribution methods themselves, residual registration or acquisition artefacts, or the model’s sensitivity to features correlated with but not causally related to the target pathology, rather than biologically meaningful localization. Recent systematic evaluations of xAI methods in neuroimaging show that commonly used attribution techniques can produce lateralized, spatially inconsistent, or non-reproducible outputs even when applied to the same model and dataset (44). Our findings are consistent with these observations. The partial overlap between CNN saliency maps and VBM findings for amyloid, tau, and CAA is encouraging and provides support for biological plausibility, but this comparison is qualitative and should not be interpreted as quantitative validation that the model has learned to detect pathology in those regions. Individual-level saliency maps should not be used as indicators of where pathology resides in a given patient, and group-averaged maps should be treated as exploratory rather than confirmatory findings.

The interpretability analyses in this study were applied to the image-only 3D CNN architecture rather than to the hybrid CNN, which achieved the strongest overall classification performance. The decision to restrict xAI analyses to the 3D CNN was deliberate as the hybrid model’s fusion of imaging and tabular data streams makes voxel-level attribution inherently ambiguous, and standard xAI methods cannot reliably disentangle the spatial contributions of MRI features from the simultaneous influence of clinical and genetic covariates. The interpretability analyses therefore characterize the imaging-derived features learned by the 3D CNN in isolation, which provides valuable insight but does not directly reflect the decision process of the best-performing multimodal model. Developing principled xAI methods for multimodal fusion architectures remains an open challenge, and extending interpretability analyses to hybrid models of this kind is a key direction for future work.

The hybrid 3D CNN-ANN architecture integrating tabular clinical/genetic data with full 3D MRI volumes consistently outperformed the other models across nearly all neuropathologies. This multimodal fusion approach likely benefits from synergistic information in both imaging and non-imaging domains while capturing spatial neuroanatomical context at high resolution. The ability of the hybrid model to perform multilabel classification further emphasizes its potential for comprehensive assessment of co-occurring pathologies, although with some limitations seen in pathologies with lower prevalence or sparser labels. 3D CNNs, though effective for volumetric data, may not adequately capture all relevant clinical variables unless combined with clinical/genetic features, as achieved in the hybrid model. Moreover, sample size and label imbalance, especially for HPSC and DLB, posed challenges across models, reflected in lower F1 scores and balanced accuracy. In summary, the integration of multimodal data using advanced deep learning architectures coupled with comprehensive explainable AI methods offers a promising avenue for non-invasive, personalized neuropathological profiling in dementia. The relatively weak F1 scores observed for hippocampal sclerosis, Lewy body pathology, and TDP-43 in multilabel settings deserve careful interpretation. These results reflect a combination of severe class imbalance, the subtler morphometric footprint of these pathologies on T1-weighted structural MRI, and increased label sparsity in the multilabel classification context. Clinicians and researchers should exercise caution in interpreting these results as evidence that deep learning can reliably detect these pathologies in clinical practice. Rather, these findings highlight specific targets where improved data collection, additional imaging modalities, and more targeted modeling will be necessary before meaningful clinical utility can be claimed. The stronger performance observed for amyloid-*β*, tau, and CAA is more encouraging and aligns with the well-established structural correlates of these pathologies on T1-weighted MRI.

A key methodological concern in studies using autopsy-confirmed labels is the temporal gap between *in vivo* imaging and postmortem assessment, during which neuropathology may continue to accumulate and introduce label noise. To explicitly evaluate this, we conducted an interval-stratified analysis of hybrid CNN performance using the median MRI-to-autopsy interval as a threshold. We did not observe a systematic degradation in classification accuracy at longer intervals. In most cases, including amyloid-β, CAA, TDP-43, and hippocampal sclerosis, performance was stable or modestly improved in the longer-interval subgroup. These findings suggest that the models are capturing relatively stable structural and clinical correlates of disease burden rather than relying solely on temporally proximate imaging features. This is consistent with the known slow accumulation of several neuropathological processes and supports the use of MRI-based models even when imaging precedes autopsy by several years. Nevertheless, residual temporal uncertainty remains a limitation, and future studies with larger autopsy-linked cohorts will be needed to support finer-grained interval modeling and longitudinal prediction.

To directly address the hypothesis that transformer-based multimodal architectures offer a superior modeling paradigm for dementia research tasks, we evaluated two representative vision transformer models within the same experimental framework as our CNN-based approaches. Contrary to expectations derived from larger clinical datasets, transformer models trained from scratch demonstrated limited and unstable performance across most neuropathological targets. This behavior is consistent with theoretical and empirical evidence that vision transformers lack strong inductive biases for spatial locality and anatomical continuity, requiring substantially larger training datasets to learn stable representations for volumetric medical imaging. For autopsy-confirmed neuropathology prediction, where sample sizes are constrained and labels are sparse, these limitations become particularly pronounced. The weaker performance of transformers in this study does not contradict recent large-cohort multimodal transformer frameworks, but highlights a critical regime distinction. Models such as those proposed by Xue et al. (56) are optimized for large, heterogeneous datasets with extensive clinical and imaging coverage, whereas pathology-validated datasets impose fundamentally different constraints. Xue et al. (56) introduced a multimodal transformer framework that integrates demographics, clinical history, neuropsychological measures, and multimodal 3D MRI to predict disease risk at scale. In contrast, our work prioritizes high-quality, research-grade 3D volumetric MRI, to attempt to infer specific neuropathologies directly from brain structure rather than relying on broad clinical feature aggregation. This focus emphasizes biological interpretability and mechanistic linkage between imaging phenotypes and the underlying pathology. Our results suggest that, under these conditions, convolutional architectures with strong spatial inductive biases may be more data-efficient, robust, and suitable for biological validation.

Transformer-based architectures represent an important and rapidly evolving direction for multimodal dementia research, particularly for integrating longitudinal clinical records, imaging, and demographic data at scale. Nevertheless, their application to autopsy-confirmed neuropathology prediction remains constrained by dataset size and label availability. A growing body of empirical work suggests that in lower-data regimes, especially in medical imaging, convolutional neural networks can outperform or match transformers due to their stronger spatial inductive biases and improved data efficiency. Additionally, while explainable AI methods for transformer-based volumetric models are emerging, the interpretability landscape remains relatively immature compared to CNN-based frameworks, with limited consensus on faithfulness and clinical reliability. In contrast, CNN-focused xAI techniques are better established in neuroimaging and enabled the present study’s systematic comparison with voxel-based morphometry. As larger autopsy-linked multimodal datasets become available, future work may examine transformer-based fusion mechanisms and hybrid architectures that retain interpretability while scaling to more complex data regimes.

A related concern is whether the deep learning models may have partially learned scanner or site-related acquisition patterns rather than pathology-specific neuroanatomical features. Explicit harmonization was not applied to the deep learning inputs. This was a deliberate choice, given evidence that harmonization can remove biologically meaningful variance relevant to classification (57–59); even so, several aspects of our design mitigate this risk. Standardized preprocessing, including intensity normalization and spatial registration, reduces low-level scanner differences prior to model input. Training on data from over 40 heterogeneous NACC sites ensures that no single acquisition pattern is consistently confounded with any pathology label. Additionally, the convergence between CNN-derived saliency maps and VBM findings, derived using an independent statistical framework that explicitly models scanner site as a covariate, supports the biological plausibility of the learned features. Nevertheless, residual scanner effects cannot be fully excluded without formal external validation, and the application of domain-adaptive modeling or prospective harmonization strategies is an important direction for future work.

## 6 Limitations

While this study offers promising advances in multimodal deep learning and explainable AI for predicting dementia-related neuropathologies, several limitations should be acknowledged. First, the models were trained and evaluated solely on the National Alzheimer’s Coordinating Center (NACC) dataset. Although this is a large, well-characterized, multi-center resource with autopsy-confirmed labels, relying on a single consortium, even though it has many sites across North America, may limit the generalizability of the findings. We did not perform a formal external validation or a rigorous site-held-out evaluation, which are considered the gold standard for assessing robustness across acquisition sites. Differences in imaging protocols, patient demographics, and disease prevalence across other cohorts could therefore impact model performance, necessitating further validation on independent datasets. A primary limitation of this study is the lack of formal external validation on an independent cohort with autopsy-confirmed neuropathological labels. Although NACC aggregates data from over 40 Alzheimer’s Disease Research Centers across the United States and therefore encompasses substantial heterogeneity in MRI acquisition protocols, scanner types, and patient demographics, this internal diversity does not substitute for validation on a fully independent dataset. To our knowledge, no other publicly available dataset currently combines matched ante-mortem structural MRI with post-mortem multi-pathology neuropathological assessment at the scale required for deep learning. As a partial proxy for external generalizability, we performed a site-held-out evaluation within NACC, which demonstrated broadly consistent performance across held-out sites. Nevertheless, differences in imaging protocols, patient demographics, and disease prevalence across external cohorts could impact model performance, and the findings of this study should be interpreted as a proof-of-concept demonstration of feasibility rather than evidence of clinical readiness. External validation on independent autopsy-linked datasets, as they become available, will be an essential next step before these models could be considered for clinical translation.

Second, the availability and distribution of neuropathological labels were uneven, and some pathologies such as hippocampal sclerosis and Lewy pathology were relatively underrepresented. Furthermore, there is considerable overlap between HPCS and TDP43 proteinopathy. This imbalance affected the classification performance and F1 scores for these conditions, reflecting challenges common in medical deep learning related to label sparsity. Future studies with larger, more balanced datasets might improve model robustness and classification accuracy, as scaling data is known to enhance deep learning performance. The MRI-to-autopsy interval of up to 15 years introduces label uncertainty that cannot be fully resolved through stratified analysis. Pathological burden continues to accumulate between imaging and death, and the degree of label noise introduced by this interval is likely to vary across pathologies depending on the rate and predictability of their accumulation trajectories. This remains an inherent limitation of studies relying on autopsy-confirmed neuropathology.

Third, the explainable AI (xAI) techniques were applied only to the 3D CNN model and not to either the AutoGluon multimodal models or the hybrid CNN. The principal reason is the complexity and architectural differences: AutoGluon’s ensemble and automated pipeline structure, and the hybrid CNN’s combination of tabular and imaging branches make consistent and reliable attribution of feature importance difficult. The integrated nature of the hybrid model makes it challenging to disentangle spatial attributions derived from MRI from the influence of clinical and genetic covariates, making voxel-level interpretations potentially misleading. Consequently, interpretability analyses were restricted to the simpler 3D CNN to isolate and validate imaging-derived signals. While xAI methods provide valuable insights into model decision-making, these techniques can face challenges such as sensitivity to noise, variability across methods, and the potential for highlighting correlated rather than causative features. Hence, caution is needed in drawing definitive biological conclusions solely from xAI maps without complementary evidence. A potentially valuable extension would be to apply model-agnostic saliency methods such as occlusion sensitivity analysis (OSA) to the AutoGluon pipeline and compare the resulting attribution maps with those derived from the 3D CNN. However, AutoGluon operates as a black-box automated ensemble combining multiple heterogeneous model types, meaning that OSA applied to AutoGluon would reflect the aggregate sensitivity of a heterogeneous ensemble rather than a coherent set of spatially learned neuroanatomical features. Furthermore, AutoGluon’s image handling treats MRI slices as generic 2D images without neuroanatomical awareness, meaning that pixel-level saliency maps derived from AutoGluon are not neuroanatomically grounded in the same way as the 3D voxel-wise maps produced by the CNN, and a principled voxel-wise neuroanatomical comparison between the two would not be straightforward. A fair cross-model OSA comparison in a shared neuroanatomical space would require both models to operate as spatially aware volumetric architectures with coherent feature extraction mechanisms. This represents a meaningful direction for future work, particularly as AutoML frameworks with native 3D volumetric and neuroanatomically aware support become more widely available. The xAI saliency maps in this study reflect model attention patterns rather than direct localization of neuropathology. Unexpected findings including lateralization and brainstem emphasis may reflect methodological limitations of attribution methods, residual registration artefacts, or scanner confounds, and should not be over-interpreted as biologically meaningful signals without independent corroboration.

Additionally, while the scanner site was explicitly modeled in the voxel-based morphometry analyses, the deep-learning models did not incorporate scanner-aware covariates or harmonization strategies. Although standardized preprocessing and multi-site training may mitigate some acquisition-related variability, explicit harmonization methods or domain-adaptive modeling were not employed and represent an important direction for future work. While the multi-site heterogeneity of the NACC training data reduces the likelihood of site-confounded learning, and while the convergence of CNN saliency maps with VBM findings supports for the biological plausibility of the learned features, we cannot exclude the possibility that the models partially captured acquisition-related variance. A formal empirical analysis of scanner effects was not feasible, as field strength information was not balanced across pathology groups, and the dataset size is insufficient to support stable subgroup analyses stratified by acquisition parameters. Future work should assess explicit scanner-aware modeling, domain adaptation, or prospective harmonization strategies to further address this limitation.

While the modeling components employed in this study build on established machine learning techniques, they are all integrated in a novel way to address a biologically verified and important task: non-invasive, *in vivo* prediction of multiple co-occurring dementia-related neuropathologies using autopsy-confirmed ground truth. By shifting the focus from diagnosis or single-biomarker prediction to multi-pathology inference, and by systematically combining multimodal learning with comparative interpretability analyses, we provide a novel approach to detect neuropathology and understand which predictors perform best. The training curves for several neuropathological classifiers show a persistent gap between training and validation accuracy, which may appear to suggest overfitting. However, the training parameters were selected based on systematic hyperparameter tuning in our prior published work on the same architecture and closely related tasks. The observed gap is more likely due to the limited size of the autopsy-confirmed dataset and the class imbalance for several neuropathological targets. Both of these factors are well-documented causes of train-validation divergence in deep learning independent of hyperparameter choices. The hybrid CNN already incorporates dropout regularization, instance normalization, and early stopping based on validation loss, and the test set performance metrics confirm meaningful generalization to held-out data. Future work with larger, more balanced autopsy-linked cohorts will help further close this gap.

Performance for hippocampal sclerosis, Lewy body pathology, and TDP-43 was limited, particularly in terms of F1 score, reflecting the combined effects of class imbalance, small positive case counts, and the subtle or diffuse morphometric signatures of these conditions on T1-weighted MRI. Confidence intervals for reported metrics were not computed, as the small test set size for these pathologies would produce wide and potentially unreliable interval estimates. Calibration analysis was similarly not feasible given test set size constraints. More detailed uncertainty quantification should be a priority in future work with larger and more balanced autopsy-linked cohorts.

Although this study focuses on T1-weighted MRI scans that capture structural information relevant to many neuropathologies, other modalities such as diffusion MRI, functional MRI, PET amyloid and tau imaging, or biofluid markers of amyloid and tau could provide additional complementary information and improve multi-pathology classification. Finally, although multimodal fusion demonstrated clear benefits, the models did not account for potential longitudinal changes in imaging or clinical features, which could further enhance prediction accuracy for progressive diseases like dementia in future work. In summary, while this work advances the integration of deep multimodal learning and explainable AI for neuropathology prediction, addressing issues of dataset diversity, richer multimodal imaging, balanced labels, and broader interpretability remains crucial for clinical translation and generalizability.

## 7 Conclusion

This study systematically evaluated several modeling approaches for the prediction of multiple dementia-related neuropathologies from multimodal data, including structural MRI, clinical, demographic, and genetic covariates. The results underscore the complementary strengths and limitations of the individual models (Random Forest, AutoGluon, 3D CNN, Hybrid CNN) and provide critical insights into the promise of xAI methods over traditional VBM approaches. They also show the potential of multimodal fusion approaches for advancing precision diagnostics in ADRD. Future work will focus on expanding to longitudinal MRI data, integrating additional imaging or biofluid modalities where available, and validating the models on external cohorts to ensure generalizability. Additionally, refining model architecture through self-supervised pretraining, transformer-based fusion mechanisms, and advanced explainability methods will allow more effective use of heterogeneous data while maintaining clinical interpretability. Overall, our study shows the potential of multi-target, multi-modal artificial intelligence approaches for neuropathology prediction, contributing to the advancement of non-invasive, patient-specific diagnostics and the broader goal of precision medicine in dementia research.

## Data Availability

Publicly available datasets were analyzed in this study. This data can be found at: https://naccdata.org.
