# Supplementary material for: Multi-modal deep learning and explainable AI for predicting multiple dementia-related neuropathologies from brain MRI, clinical, and genetic data
Source: Front Neurol. 2026 Jul 27;17:1839071. doi: 10.3389/fneur.2026.1839071 (PMC13453829; doi:10.3389/fneur.2026.1839071)
Supplement: Supplementary file 1 [file Data_Sheet_1.PDF]

Data Imbalance

	Train Dataset		Validation Dataset		Test Dataset	
	Positive	Negative	Positive	Negative	Positive	Negative
Amyloid	502	113	79	7	58	22
CAA	99	215	19	24	10	33
TDP-43	103	265	17	23	14	33
DLB	230	393	40	38	27	50
HPSC	67	524	9	68	6	78
Tau (Braak)	342	197	67	36	71	38

Classification Performance of the Hybrid CNN

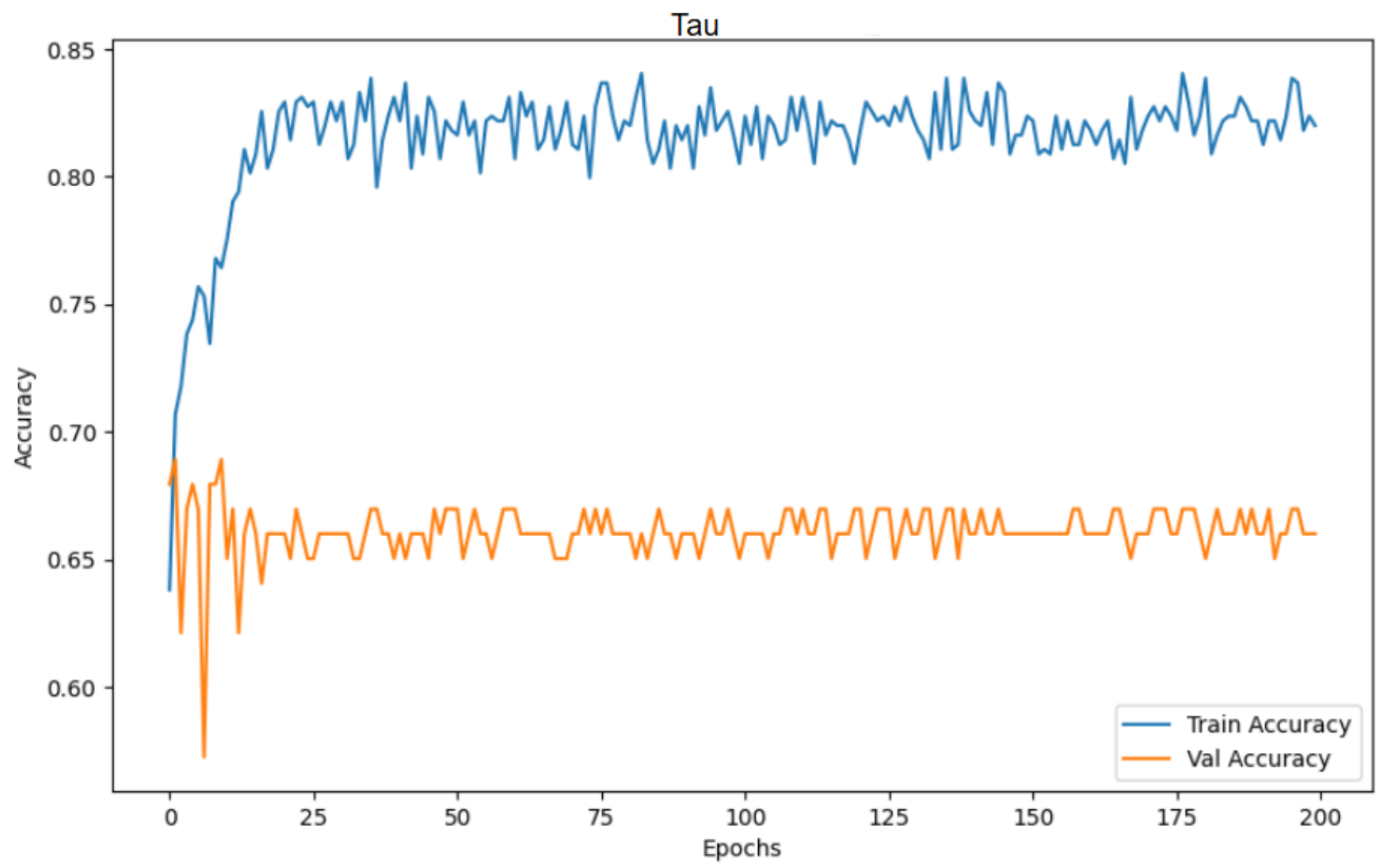

(a)

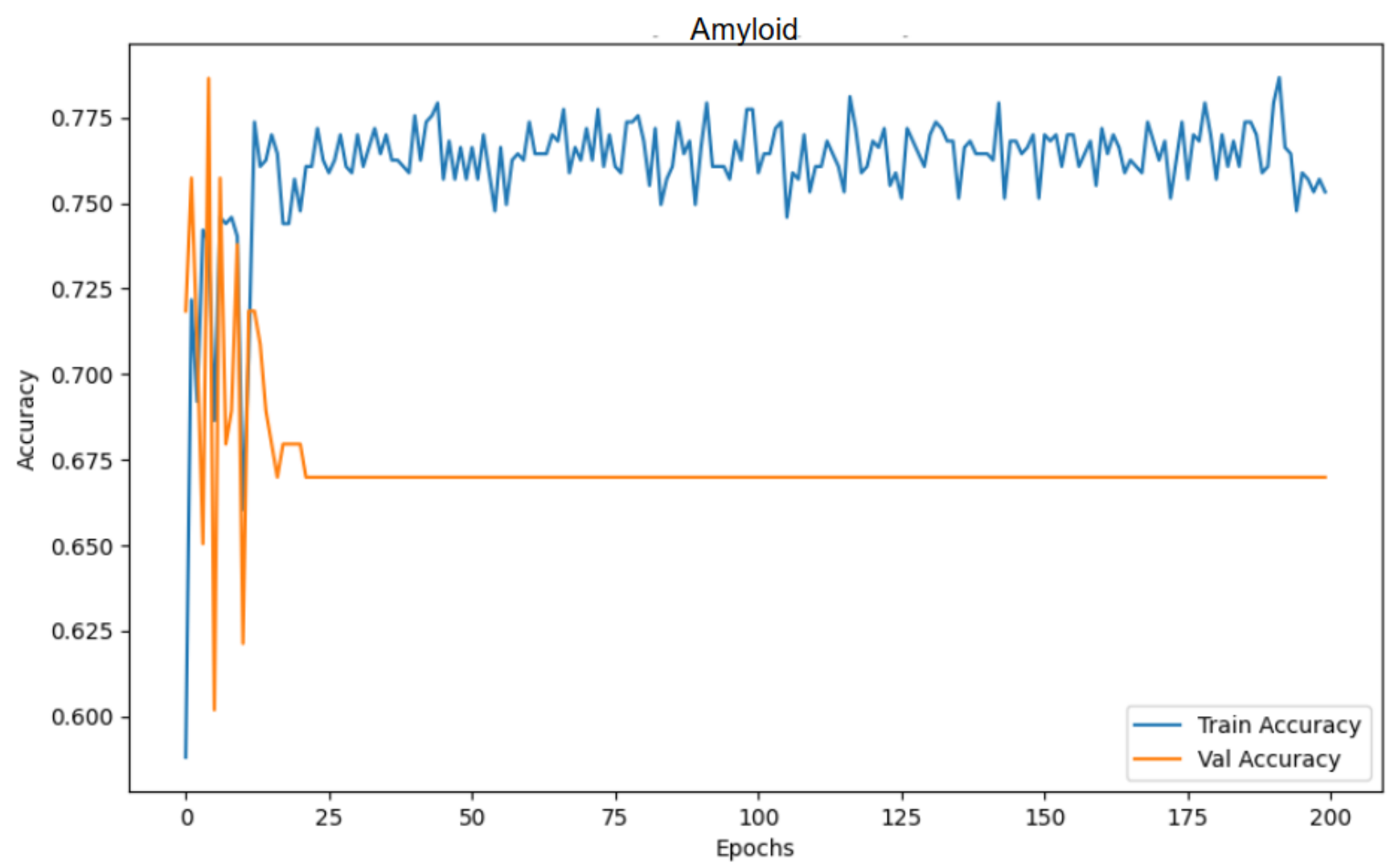

(b)

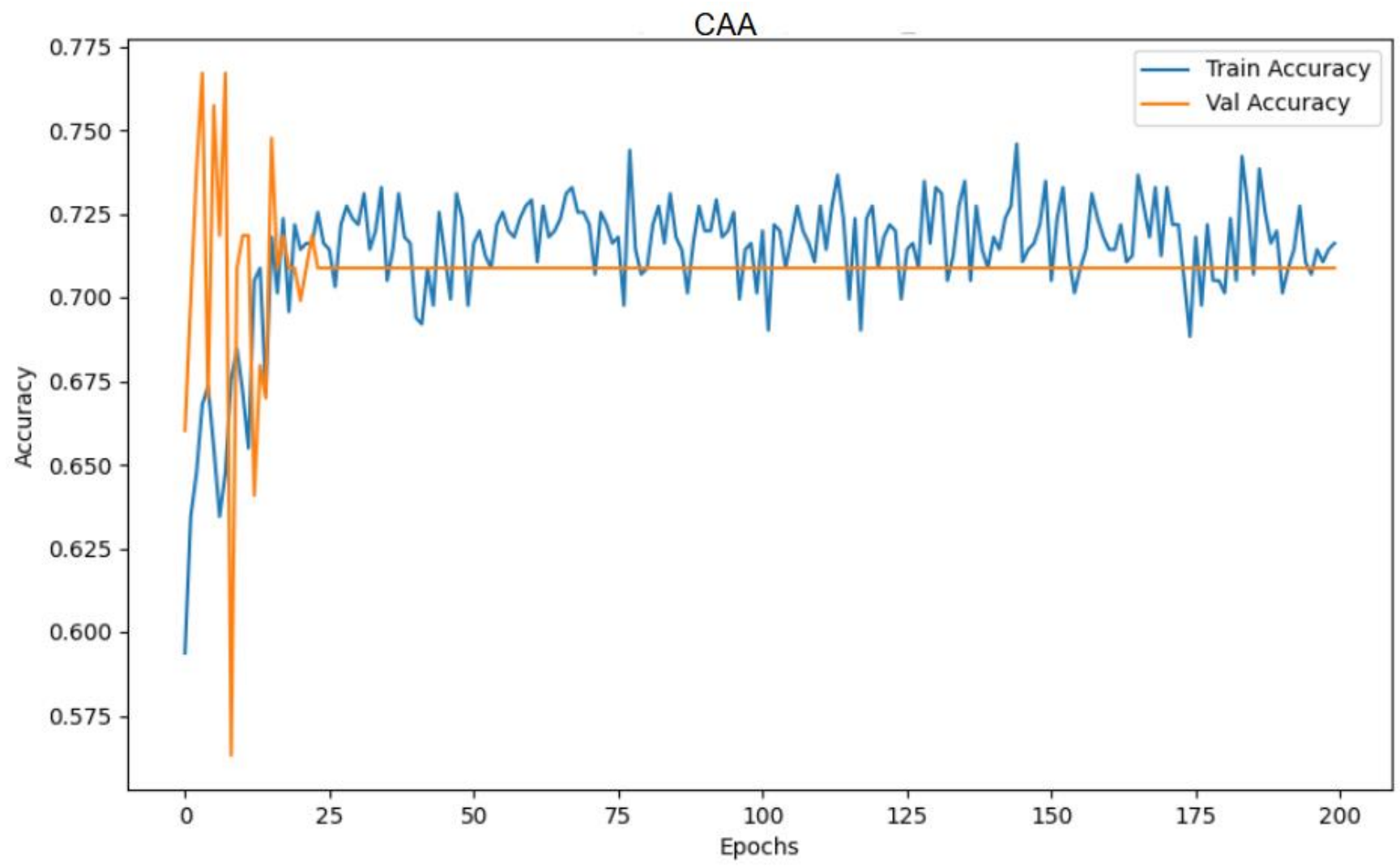
CAA
Train Accuracy
Val Accuracy
Accuracy
Epochs
0.775
0.750
0.725
0.700
0.675
0.650
0.625
0.600
0.575
0
25
50
75
100
125
150
175
200

(c)

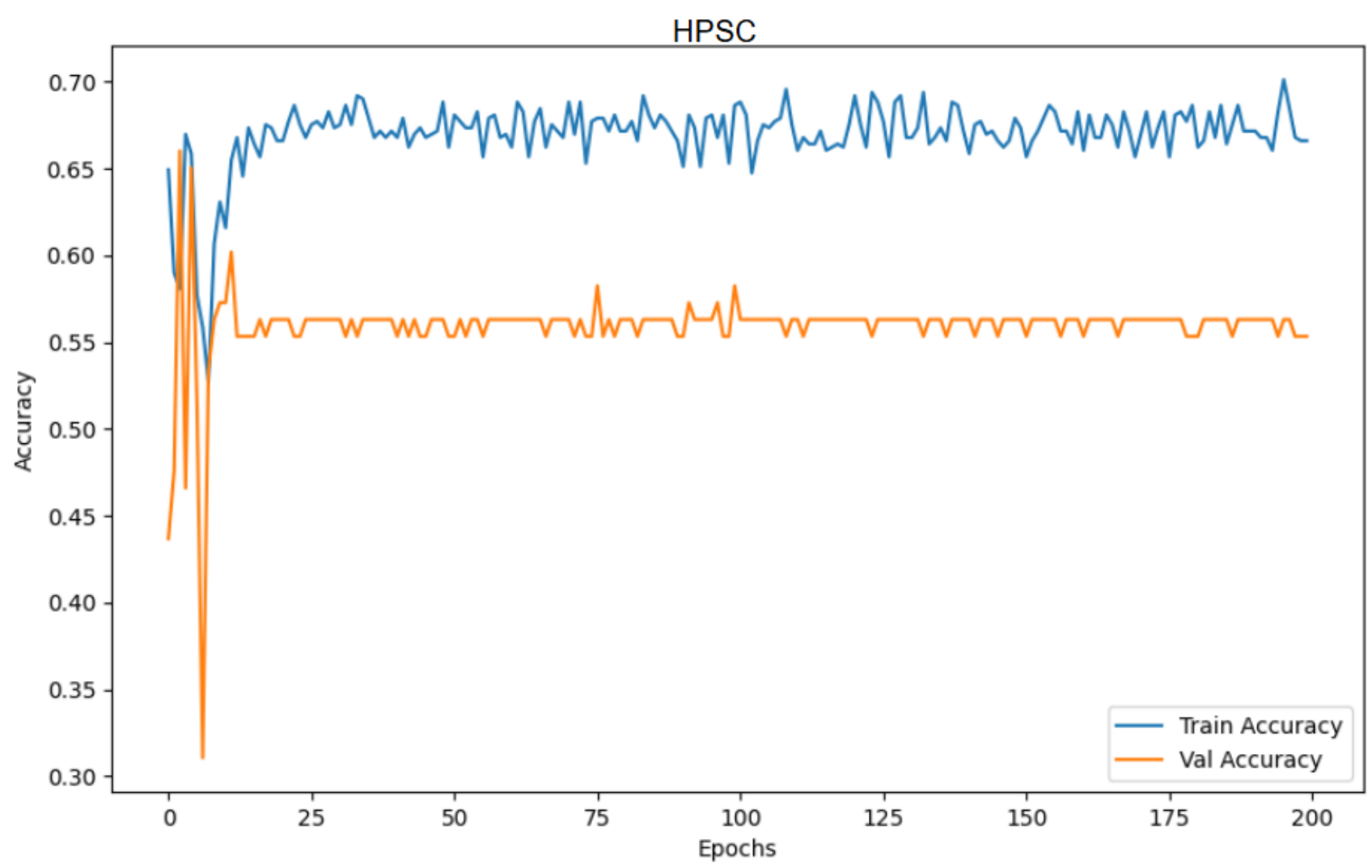
HPSC
Train Accuracy
Val Accuracy
Accuracy
Epochs
0.70
0.65
0.60
0.55
0.50
0.45
0.40
0.35
0.30
0
25
50
75
100
125
150
175
200

(d)

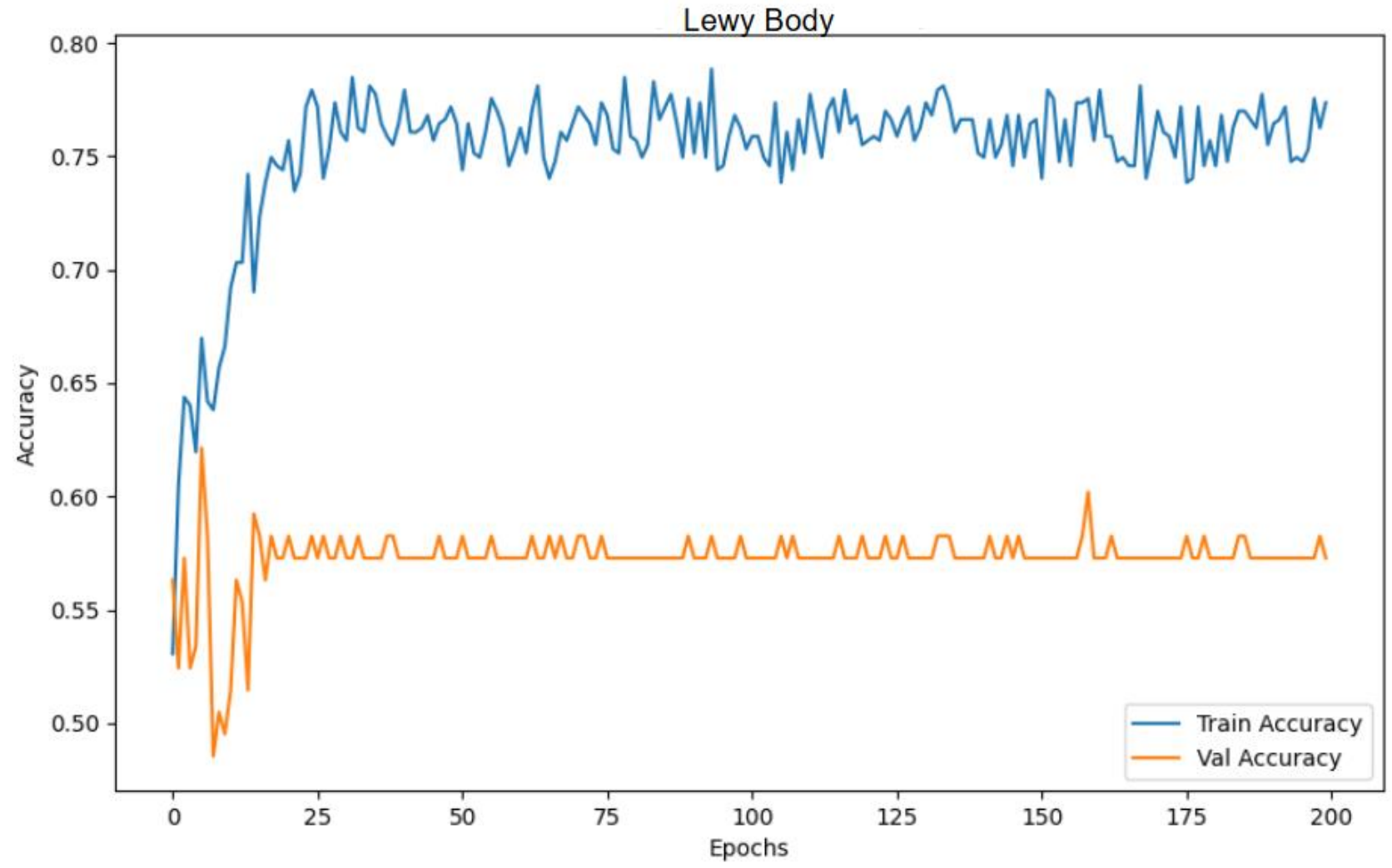

(e)

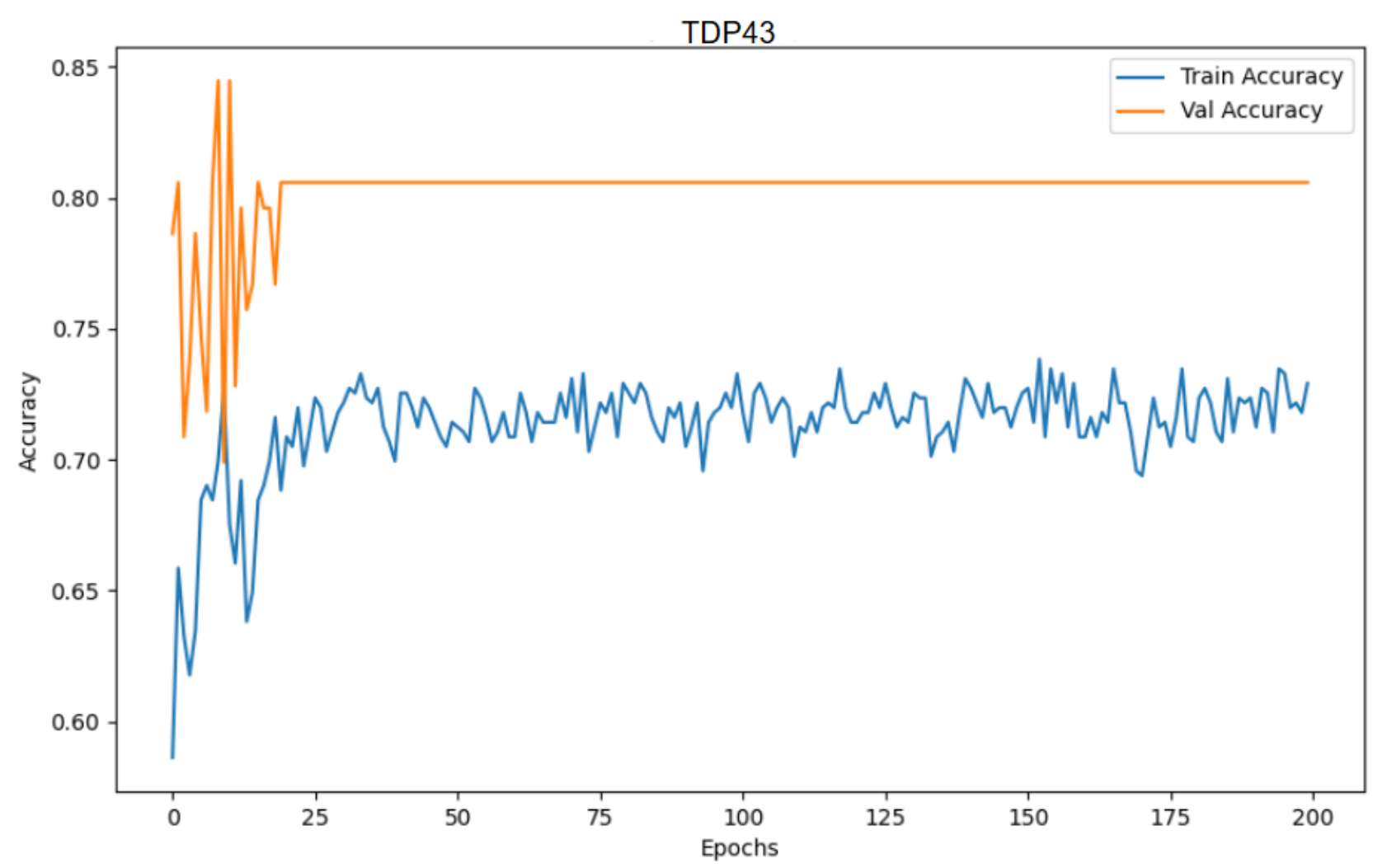

(f)

Figure 1. Training and validation accuracy curves for the hybrid CNN classifiers across neuropathological targets. Accuracy stabilizes after early training epochs, with a consistent gap between training and validation accuracy reflecting class imbalance and the use of regularization and early stopping. For the TDP-43 classification task, validation accuracy consistently exceeds training accuracy, reflecting the increased difficulty of this task and the limited sample size, which can result in higher variance and reduced stability of training performance relative to validation performance.

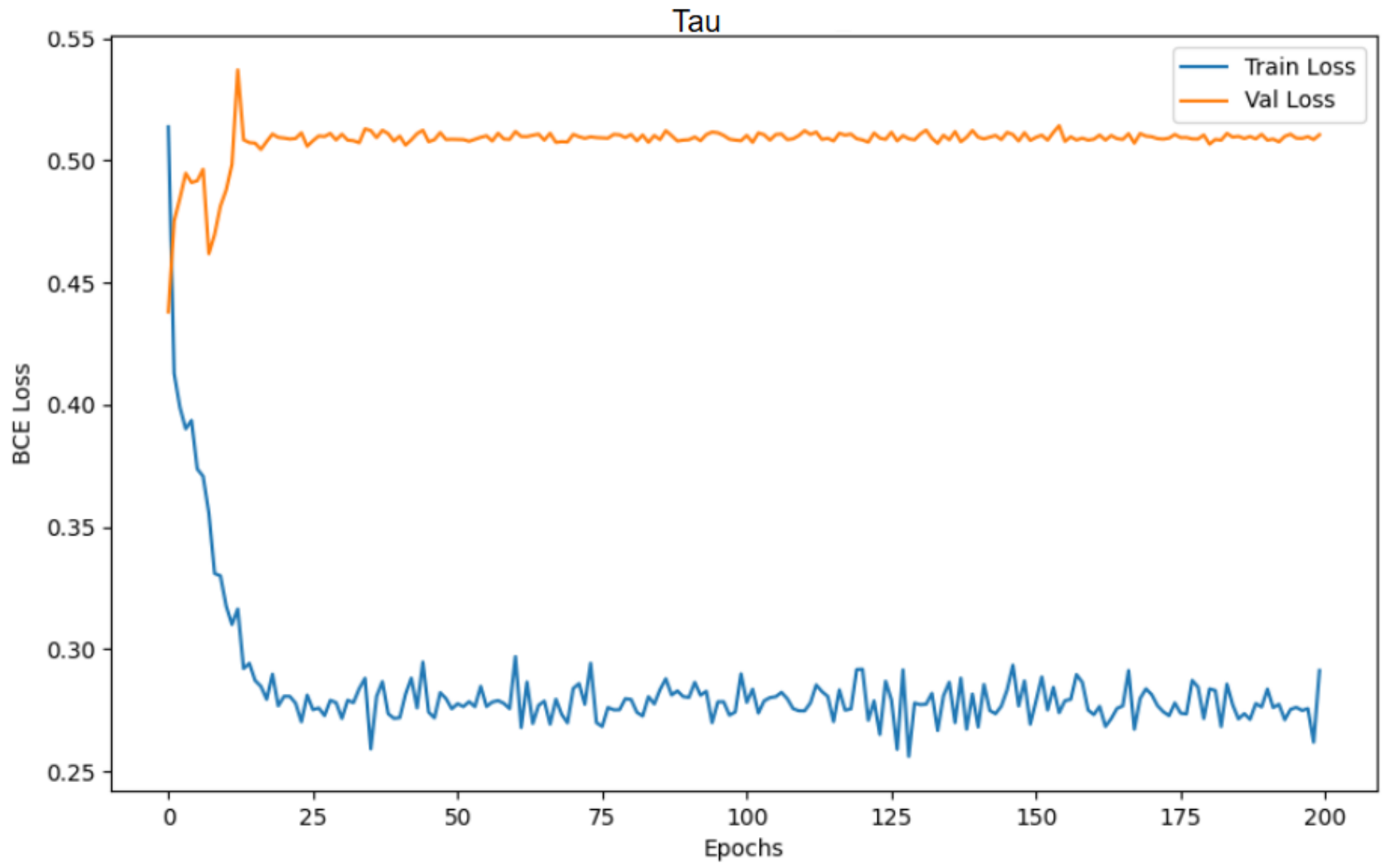
Tau
Train Loss
Val Loss
BCE Loss
0.55
0.50
0.45
0.40
0.35
0.30
0.25
0
25
50
75
100
125
150
175
200
Epochs

(a)

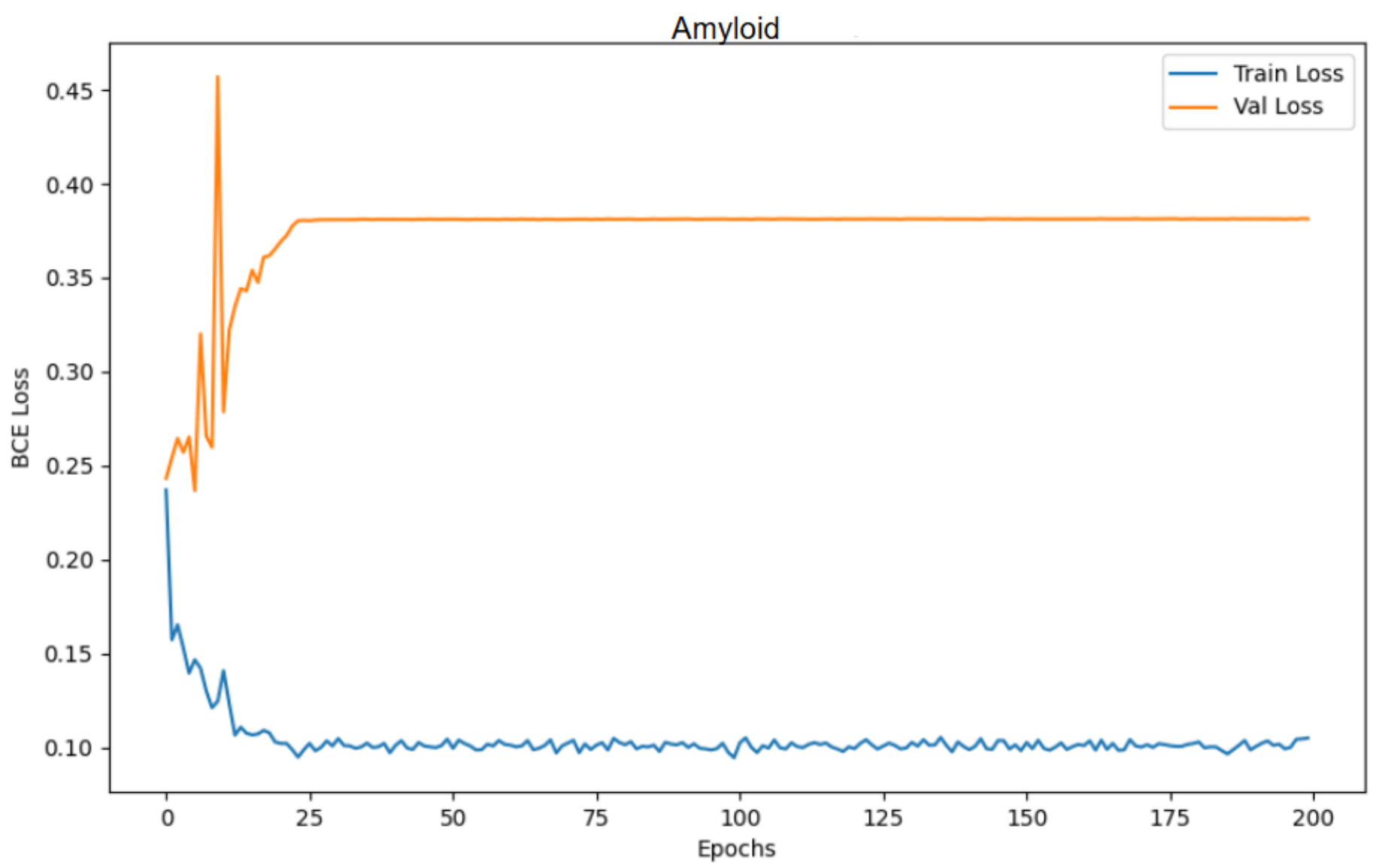
Amyloid
Train Loss
Val Loss
BCE Loss
0.45
0.40
0.35
0.30
0.25
0.20
0.15
0.10
0
25
50
75
100
125
150
175
200
Epochs

(b)

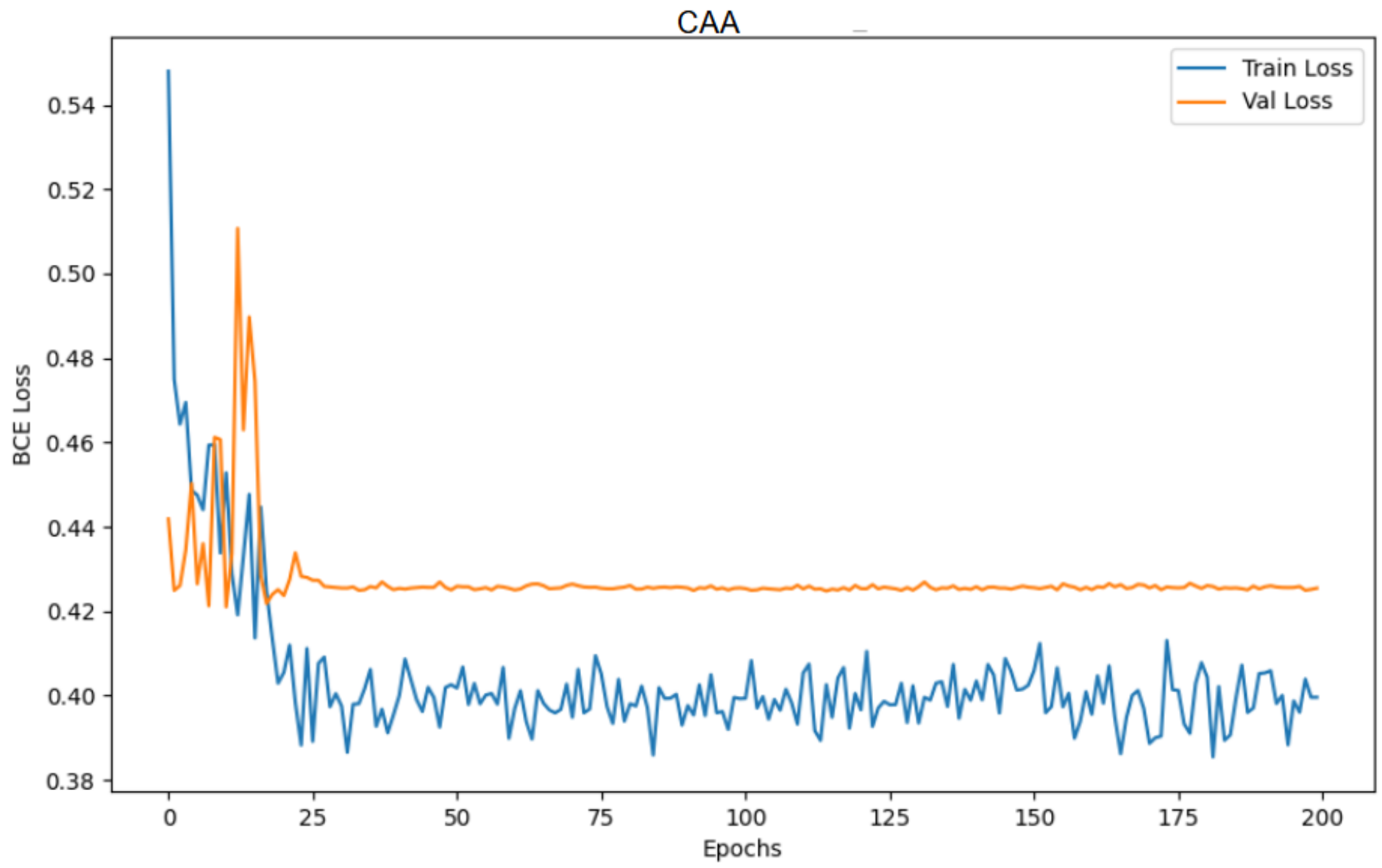
CAA
Train Loss
Val Loss
BCE Loss
0.54
0.52
0.50
0.48
0.46
0.44
0.42
0.40
0.38
0
25
50
75
100
125
150
175
200
Epochs

(c)

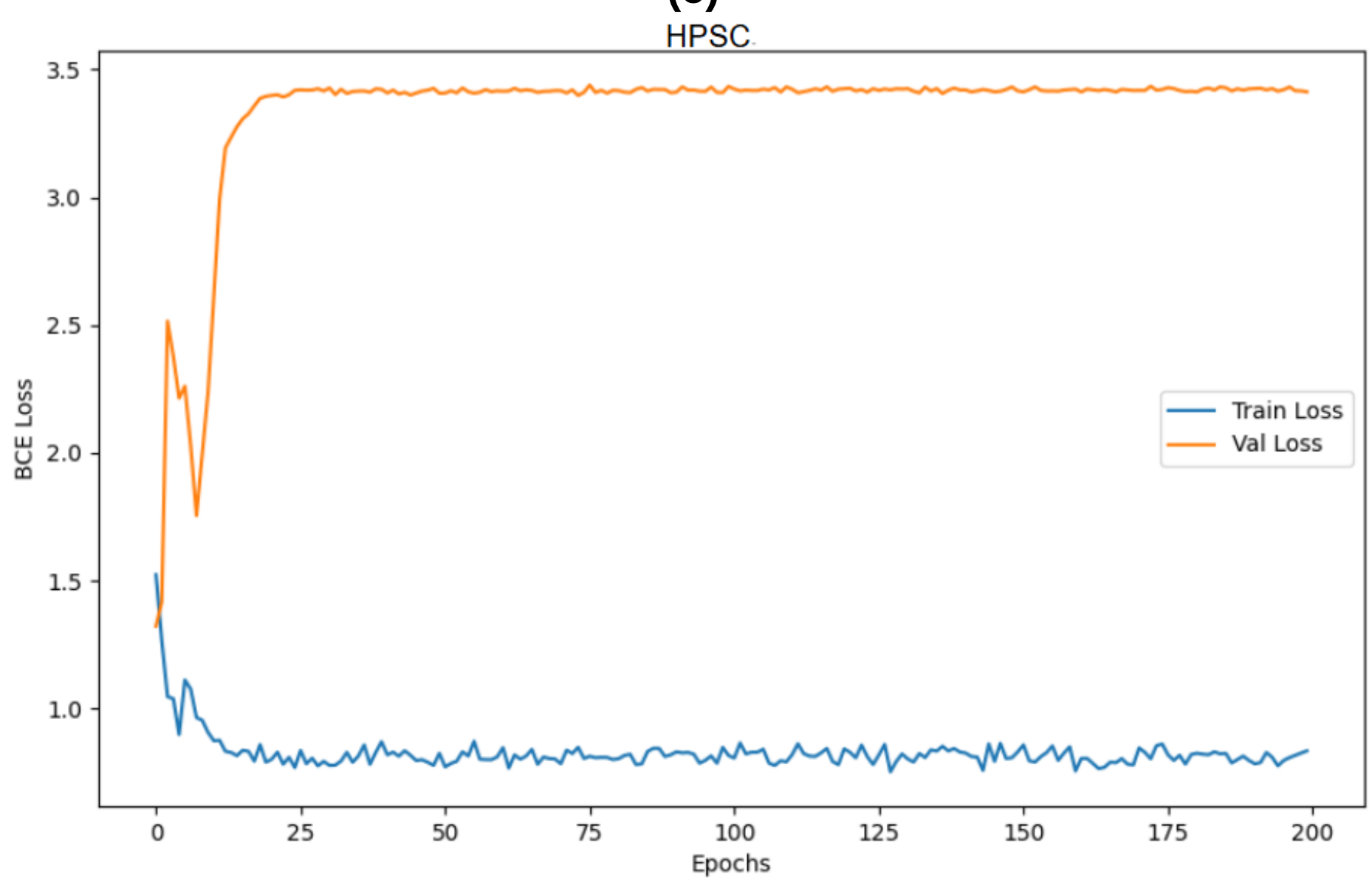
HPSC
Train Loss
Val Loss
BCE Loss
3.5
3.0
2.5
2.0
1.5
1.0
0
25
50
75
100
125
150
175
200
Epochs

(d)

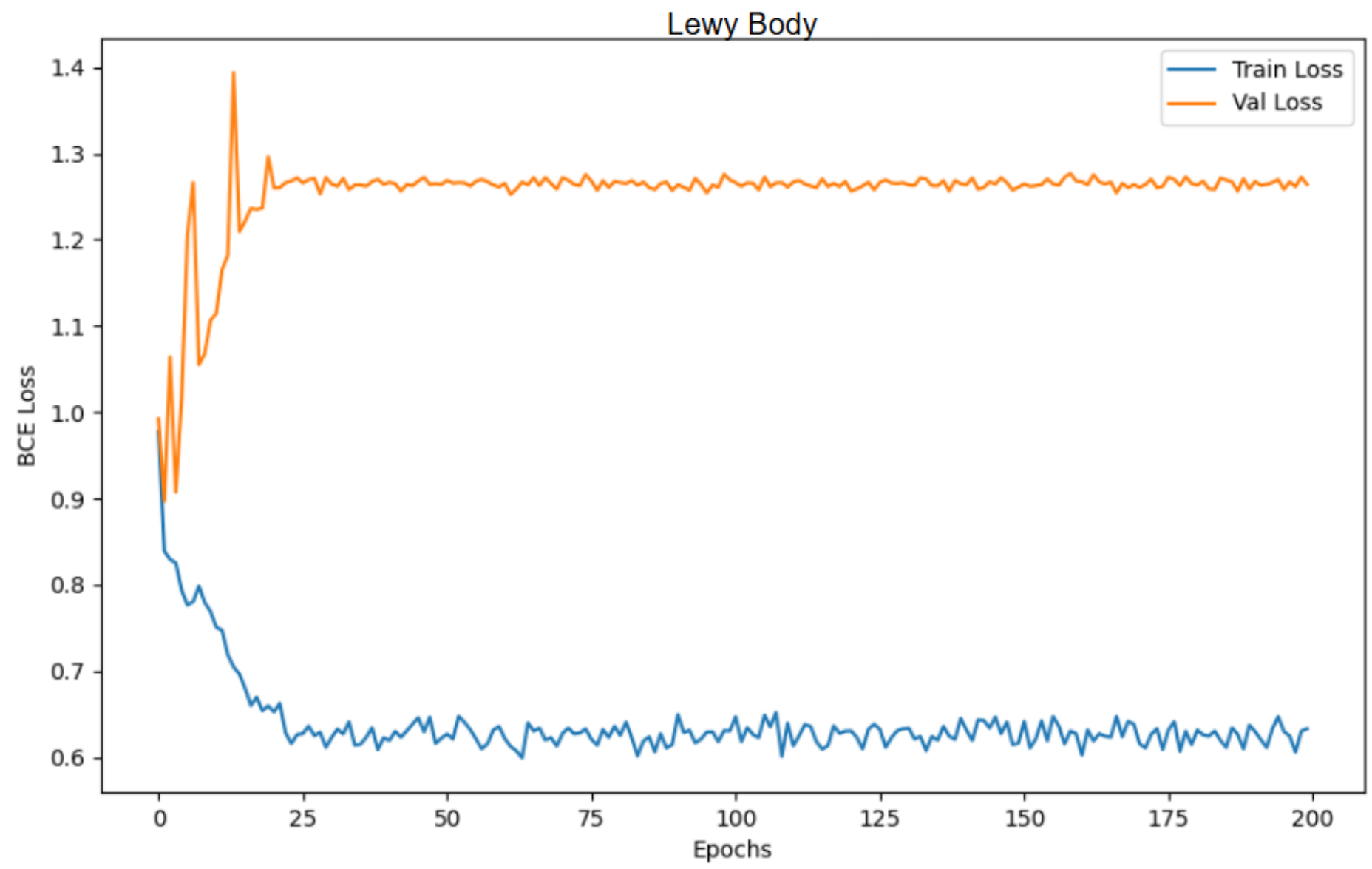

(e)

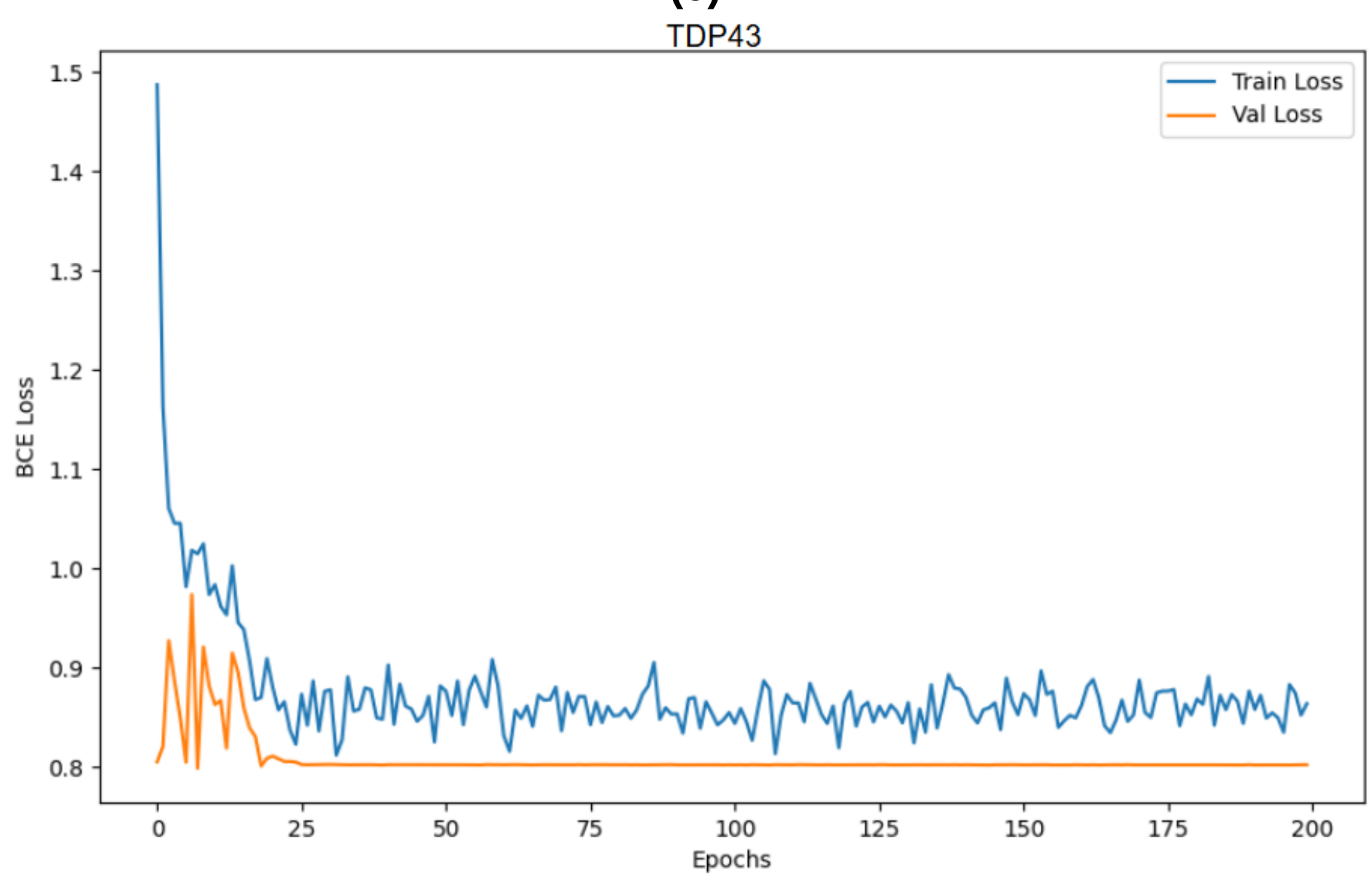

(f)

Figure 2. Training and validation loss curves for the hybrid CNN architecture across individual neuropathology classifiers. Loss values are shown as a function of training epoch. In all cases, training loss decreases rapidly during early epochs and stabilizes, while validation loss plateaus, indicating stable convergence and no evidence of severe overfitting.

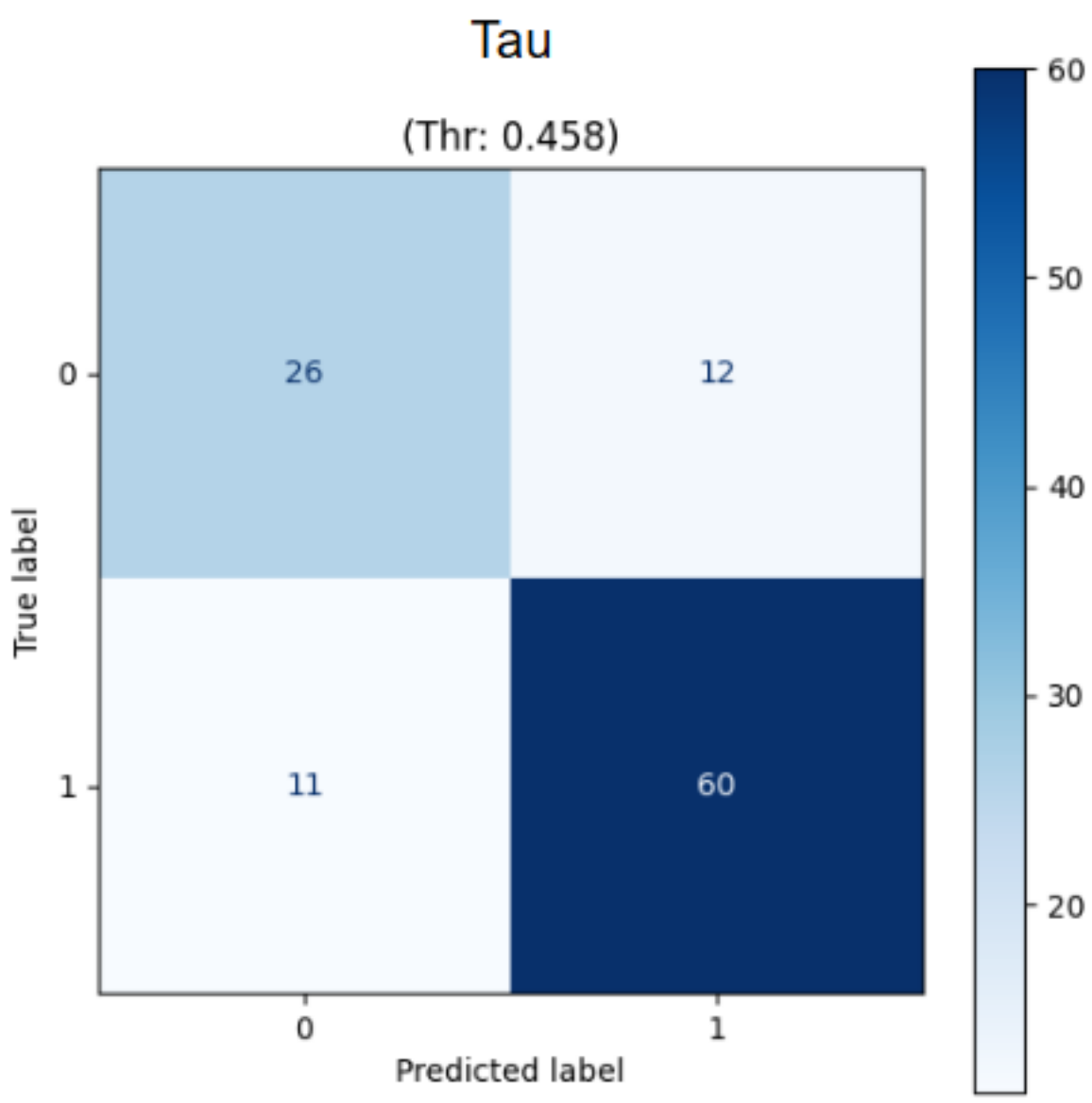
Tau
(Thr: 0.458)
26
12
11
60
True label
Predicted label
0
1
60
50
40
30
20
(a)

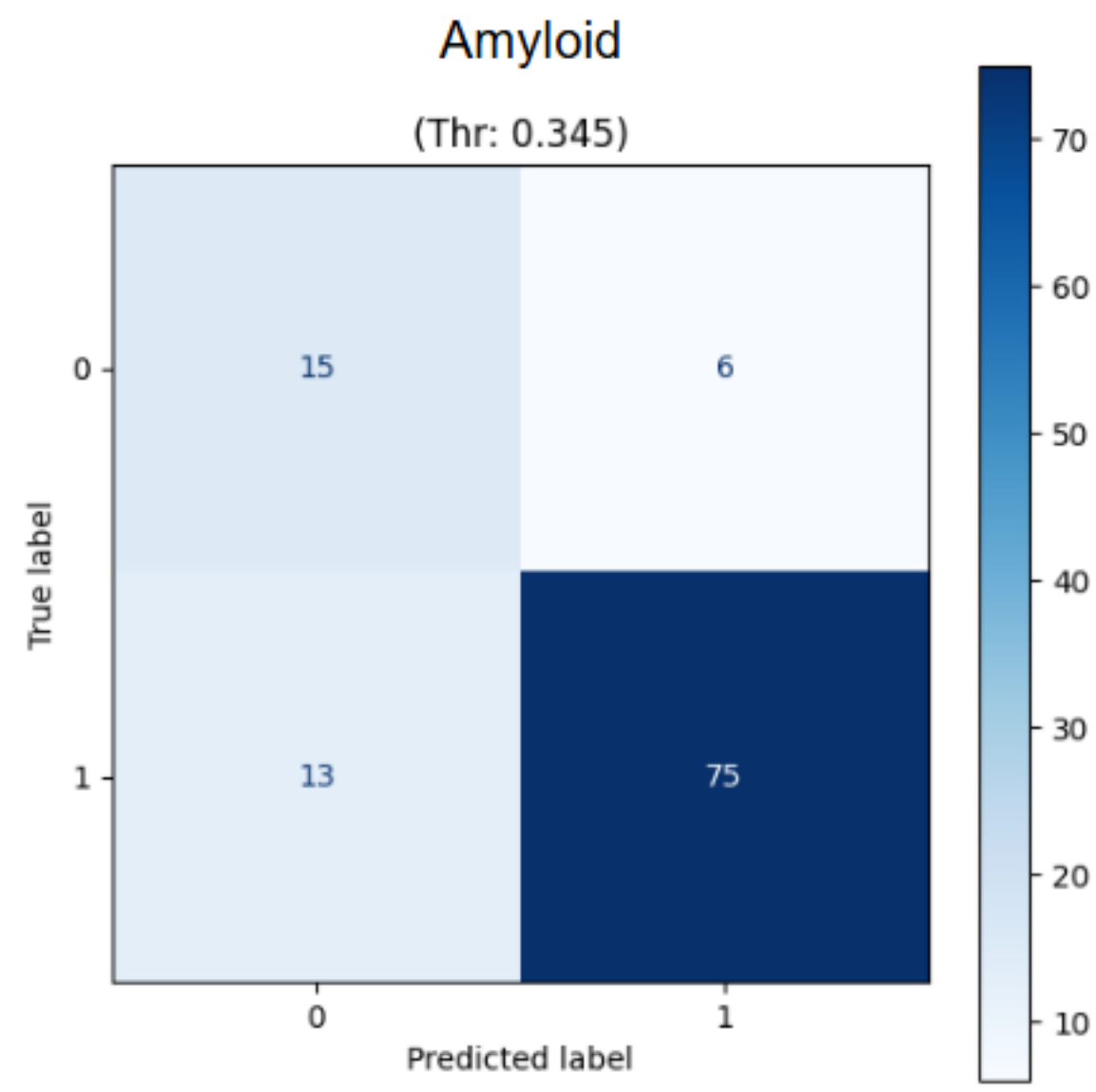
Amyloid
(Thr: 0.345)
15
6
13
75
True label
Predicted label
0
1
70
60
50
40
30
20
10
(b)

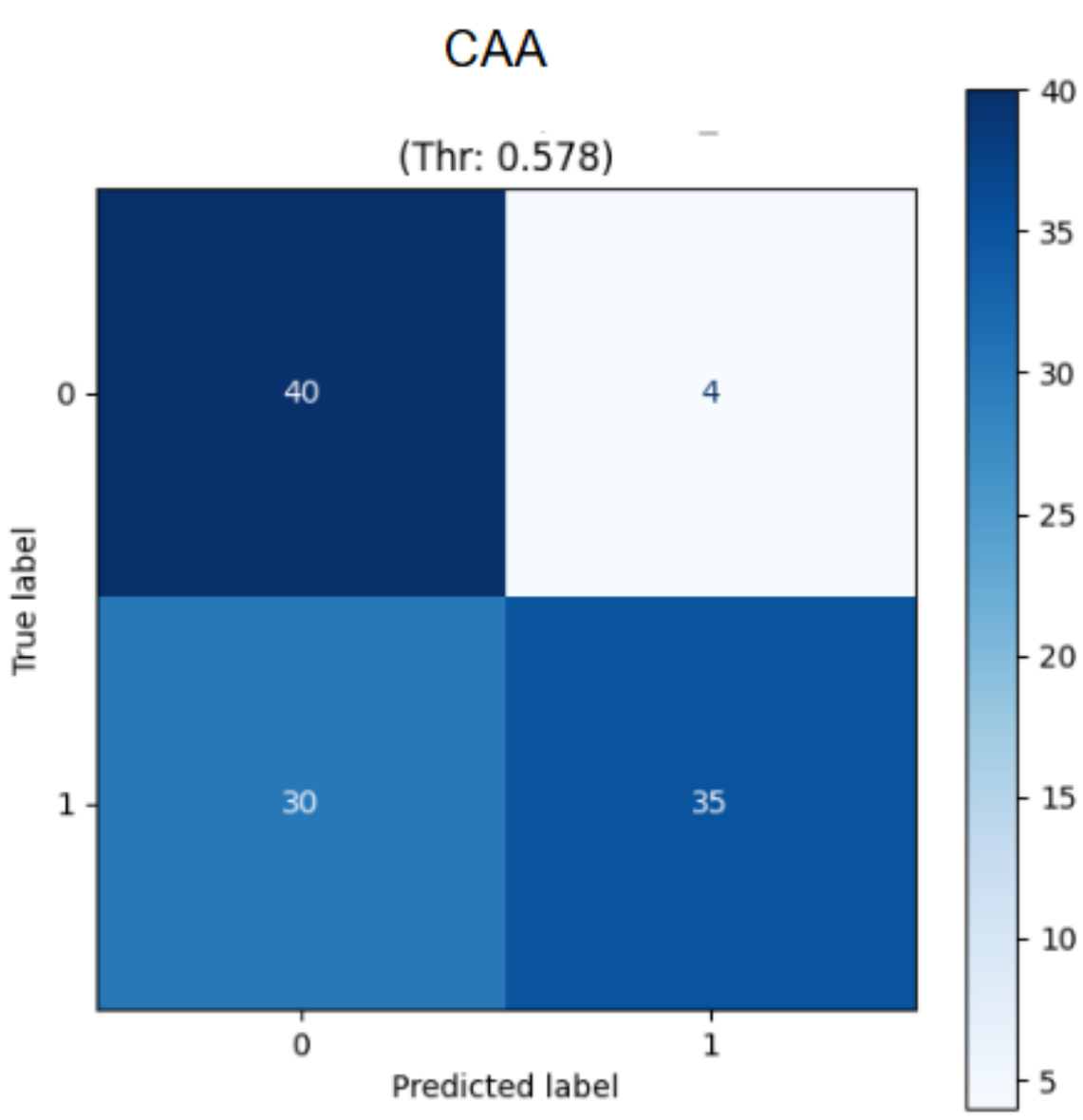
CAA
(Thr: 0.578)
40
4
30
35
True label
Predicted label
0
1
40
35
30
25
20
15
10
5
(c)

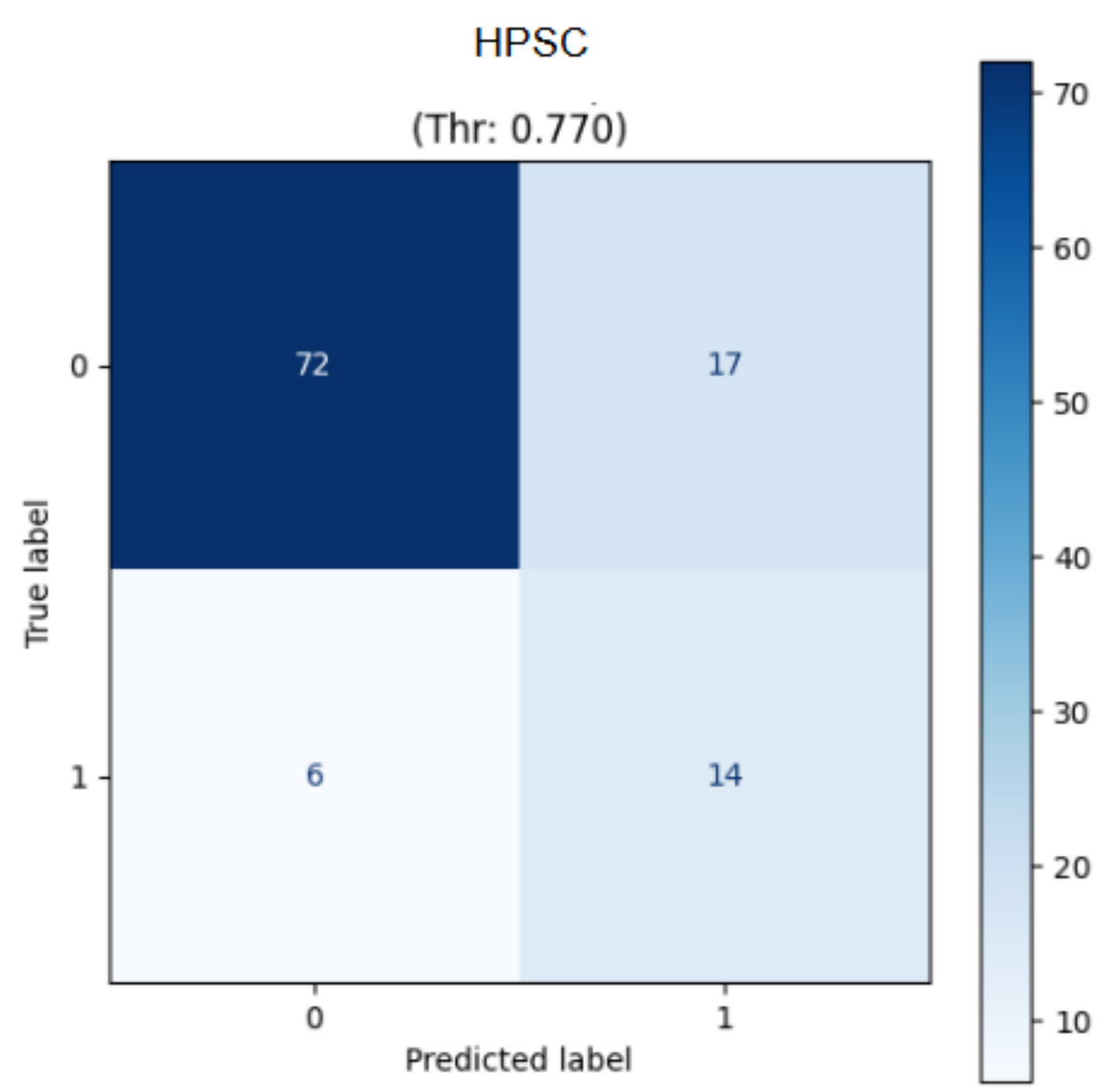
HPSC
(Thr: 0.770)
72
17
6
14
True label
Predicted label
0
1
70
60
50
40
30
20
10
(d)

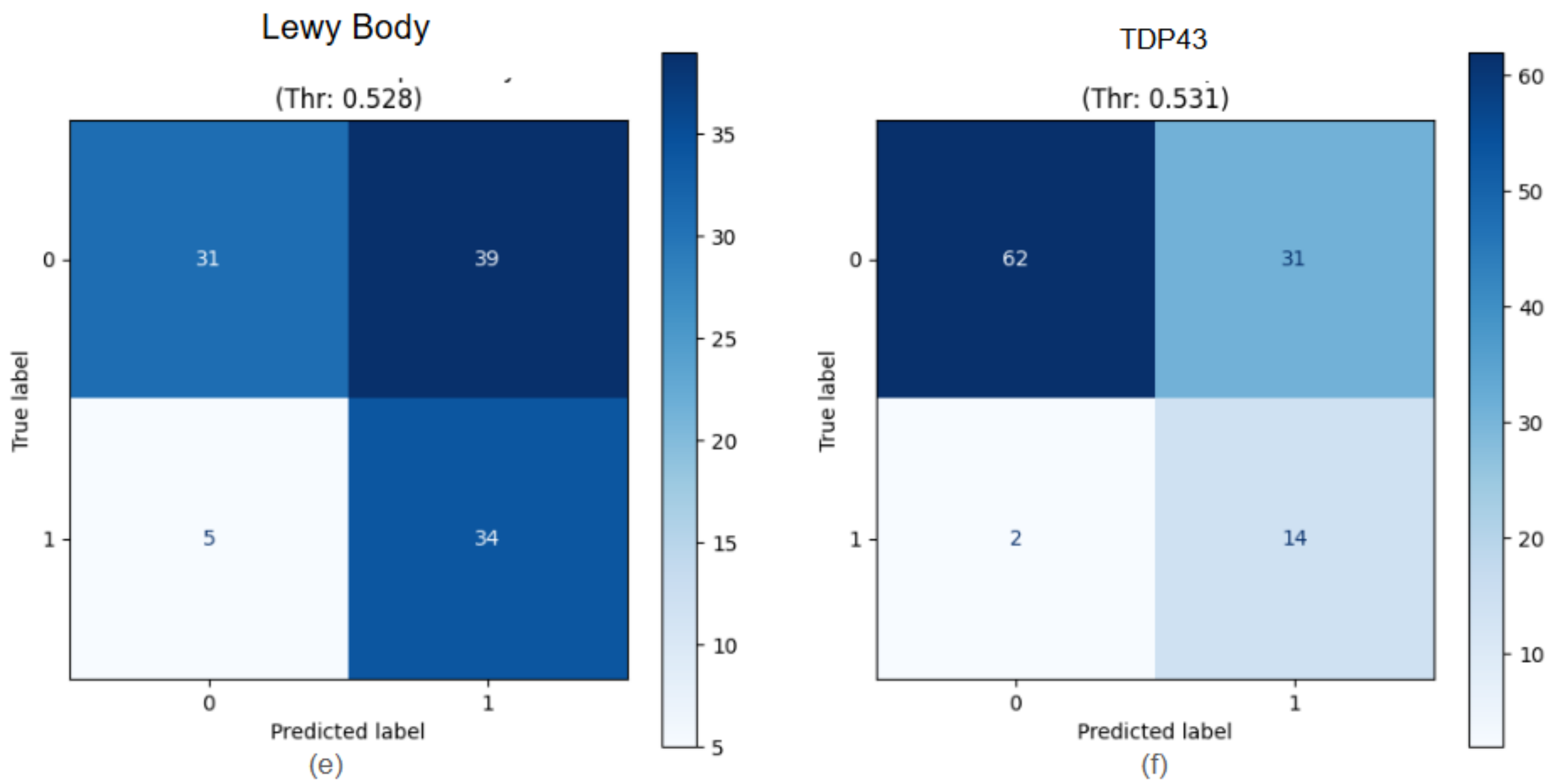

Figure 3. Confusion matrices for the hybrid CNN model evaluated on the held-out test set for each neuropathology. Matrices illustrate true versus predicted class labels using pathology-specific probability thresholds. Error patterns reflect underlying class imbalance and varying prevalence across neuropathologies.

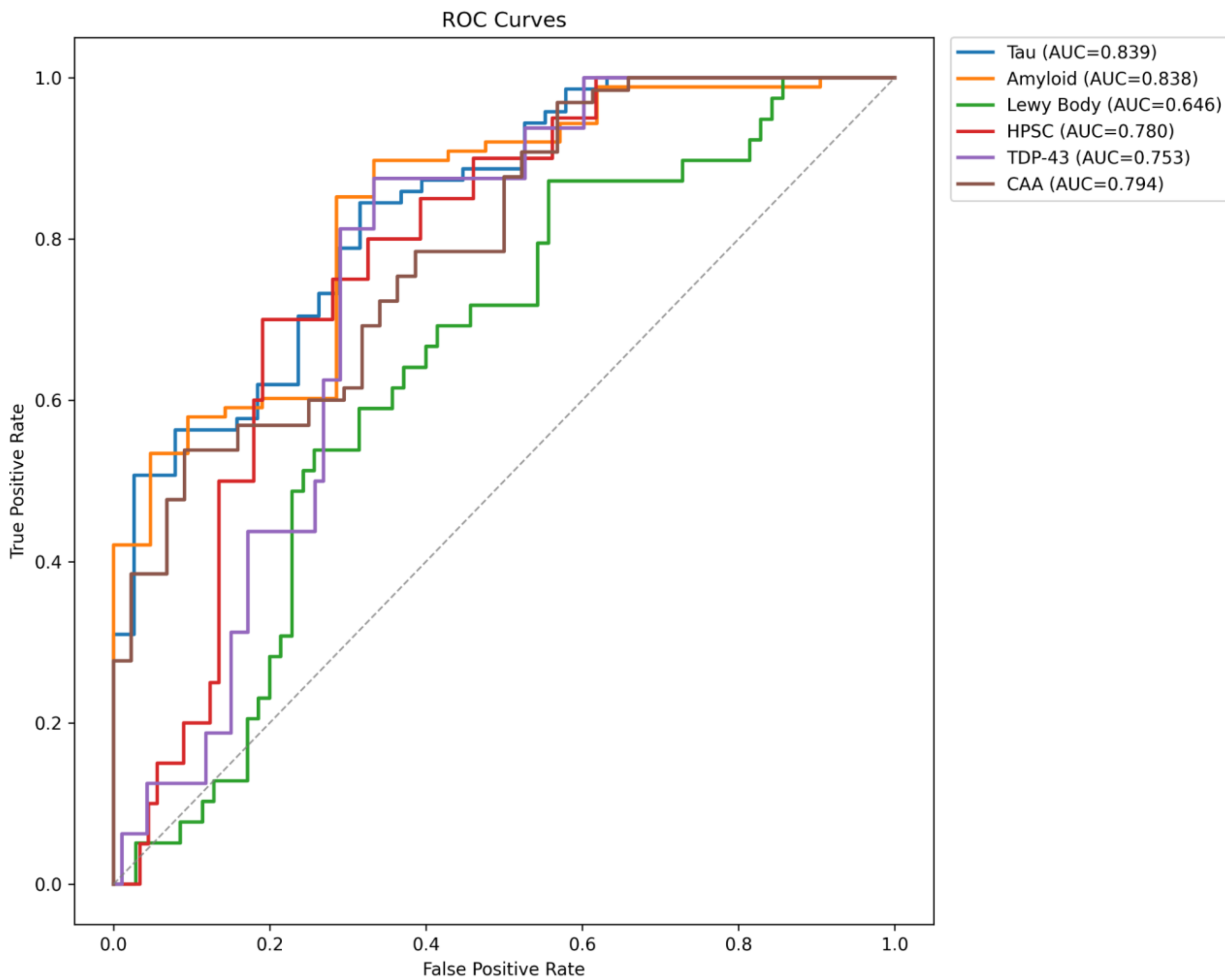

Figure 4. Receiver operating characteristic (ROC) curves for the hybrid CNN classifiers across neuropathologies, with corresponding area under the curve (AUC) values. ROC–AUC provides a threshold-independent assessment of discriminative performance and complements balanced accuracy and F1 score metrics.

To better understand the behavior of the hybrid CNN classifiers, we examined standard training and evaluation diagnostics, including learning curves, confusion matrices, and ROC-AUC plots. Figures 1 and 2 show the training and validation loss and accuracy curves for each neuropathology classifier. Across targets, training loss decreased rapidly during early epochs and stabilized thereafter, while validation loss and accuracy plateaued, indicating stable convergence without evidence of severe overfitting. Figure 3 shows confusion matrices computed on the held-out test set, illustrating class-specific prediction behavior and error patterns. These matrices highlight the impact of class imbalance for certain neuropathologies, particularly hippocampal sclerosis and TDP-43, where false negatives are more frequent despite reasonable balanced accuracy. Receiver operating characteristic (ROC) curves and corresponding area under the

curve (AUC) values are shown in Figure 4. Across neuropathologies, the hybrid CNN shows good discriminative ability, with consistently strong AUC values for amyloid-β and tau, and moderate AUCs for CAA, Lewy body pathology, and TDP-43. These threshold-independent results complement the balanced accuracy and F1 metrics reported earlier and support the robustness of the hybrid CNN framework.
